# Synthesis and properties of anhydrous rare-earth phosphates, monazite and xenotime: a review

**DOI:** 10.1039/d4ra01142b

**Published:** 2024-06-13

**Authors:** Saehwa Chong, Brian J. Riley, Xiaonan Lu, Jincheng Du, Thiruvillamalai Mahadevan, Vinay Hegde

**Affiliations:** a Pacific Northwest National Laboratory Richland WA 99354 USA saehwa.chong@pnnl.gov brian.riley@pnnl.gov +1-509-375-2469 +1-509-372-4651; b University of North Texas Denton TX 76203 USA; c Citrine Informatics Redwood City CA 94063 USA

## Abstract

The synthesis methods, crystal structures, and properties of anhydrous monazite and xenotime (REPO_4_) crystalline materials are summarized within this review. For both monazite and xenotime, currently available Inorganic Crystal Structure Database data were used to study the effects of incorporating different RE cations on the unit cell parameters, cell volumes, densities, and bond lengths. Domains of monazite-type and xenotime-type structures and other AXO_4_ compounds (A = RE; X = P, As, V) are discussed with respect to cation sizes. Reported chemical and radiation durabilities are summarized. Different synthesis conditions and chemicals used for single crystals and polycrystalline powders, as well as first-principles calculations of the structures and thermophysical properties of these minerals are also provided.

## Abstract 2

The synthesis methods, crystal structures, and general properties of anhydrous monazite and xenotime (REPO_4_) crystalline materials are summarized within this review.

## Graphical abstract

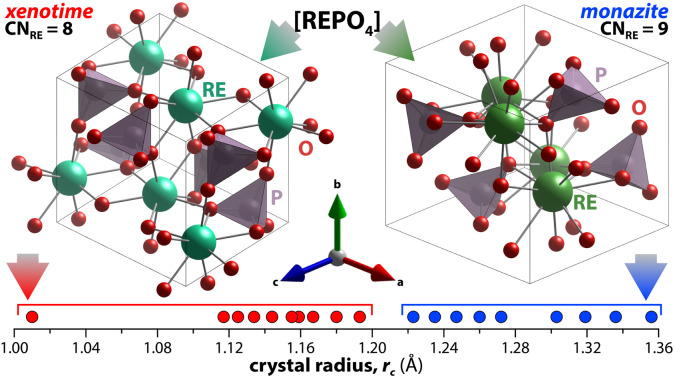

## 1 Introduction

Monazite and xenotime compounds are anhydrous rare-earth (RE) phosphates with the chemical formula REPO_4_ but with different crystal symmetries. The mineral name “monazite” is derived from “monazein” meaning “to be solitary” in Greek, and the mineral name “xenotime” is derived from the combined words of “xenos” and “time” meaning “foreign” and “honor” in Greek.^1^ Naturally occurring monazite and xenotime minerals are often found as accessory minerals along with allanite, sphene, fluorite, and apatite in granitic rocks, pegmatites, carbonatites, and gneisses.^1,2^ The monazite and xenotime minerals found in nature often contain mixed RE elements as well as thorium and/or uranium along with other oxides, and the deposits are found in various countries including Australia, Brazil, Canada, China, India, Italy, Madagascar, Sri Lanka, and the United States.^1,3–5^ For example, the compositions of natural monazites from seven different regions were 35–65 mass% of mixed REs, 24–30 mass% of P_2_O_5_, 0–16 mass% of UO_2_, 4–14 mass% of ThO_2_, and 2–7 mass% of other oxides, and among the mixed REs, La, Ce, and Nd were the major components.^2,3,6^ The natural xenotime mineral generally contains mixed heavy REs (*i.e.*, RE = Gd → Lu + Y, Sc) and less actinides compared to monazite (*i.e.*, RE = La → Dy). The compositions of natural xenotime from two regions were 62–65 mass% of mixed RE oxides, 29–36 mass% of P_2_O_5_, 0.1–1.6 mass% of UO_2_, 0.2–0.3 mass% of ThO_2_, and 0.1–1.3 mass% of other oxides, and among the mixed REs, Y, Gd, Dy, and Er were the major components.^7,8^ Rhabdophane minerals are hydrated RE-phosphates with the chemical formula of REPO_4_·*x*H_2_O and they are found as accessory minerals in granitic rocks. Rhabdophane minerals generally contains mixed REs with relatively high amounts of Ce and La elements, U and Th actinides, and one water molecule (7–8 mass%) per formula unit.^9^ Studies have shown that the rhabdophane structure can transform to the monoclinic monazite structure through heat treatments.^10,11^

Both monazite and xenotime compounds have attractive physical and chemical properties over a wide range of applications including nuclear waste forms,^12–20^ light emitting materials (*e.g.*, as scintillators for γ-ray or X-ray detection, as thermophosphors),^1,21–25^ and coating materials.^26–28^ For applications in nuclear waste forms, the REPO_4_ compounds have been studied extensively due to high chemical durabilities, resistance to radiation damage, a wide variety of natural analogs, and their potential to incorporate RE, U, and Th elements in the structures.^12–15,17,29^ The flexibility of the RE–O bond distances in REO_*x*_ polyhedra, while maintaining the structure, enables the incorporation and substitution of different heavy radioactive cations. This possibility of monazite and xenotime to incorporate many different radionuclides into a single structure makes it an ideal waste form that eliminates the need to determine the radionuclide partition coefficients between phases and concerns for differences in mechanical properties and corrosion mechanisms.^12^ Both monazite and xenotime can be synthesized relatively easily (see Section 2 for details), and their chemical durabilities are much higher (up to a factor of 20 in leach rates for certain elements) than borosilicate glass waste forms.^12^ Natural monazite was shown to maintain high retention of fission products during erosion, which was attributed to its slow dissolution rate.^13^ In addition, both compounds have high thermal stabilities with melting temperatures above 2000 °C.^12^

Monazite compounds can be used as light emitting materials with interesting optical properties.^21–23^ Wang *et al.*^21^ investigated the luminescence behavior of Eu-doped LaPO_4_ nanorods and showed the emission spectrum dependency on polarization for electric and magnetic dipole transitions. With high quantum efficiency and a high degree of polarization, the Eu-doped LaPO_4_ nanorods can be used as high resolution probes in 3D flow-shear tomography.^21^ Hashimoto *et al.*^22^ observed that doping a small amount of Th and borate into monazite compounds containing mixed RE cations of La, Ce, and/or Tb improved the light emission intensity and stability of green light, and these phosphors used in fluorescent lamps can minimize the brightness loss at high temperatures. Jeon *et al.*^23^ doped La- and Ca-containing monazite compounds with Eu and/or Dy ions, and single-phase white-light emission was observed at near-ultraviolet excitation.

The LaPO_4_ monazite has also been explored as a coating material to minimize high-temperature oxidation.^26–28^ Morgan *et al.*^27^ showed that LaPO_4_ and alumina interfaces without impurities were stable and retained the ability to debond after heat treatment at 1600 °C in air but observed the formation of La-containing β-alumina-magnetoplumbite in the presence of alkali metal or divalent elements near the interface. Kuo and Kriven^26^ showed that three laminates composed of LaPO_4_ as one component and Al_2_O_3_, Y_3_Al_5_O_12_, or LaAl_11_O_18_ as the other component were thermally stable up to 1600 °C and could be used as high-temperature materials in an oxidizing environment. Boakye *et al.*^28^ showed that coating the SiC fibers with LaPO_4_ did not degrade fiber strength, and a heat treatment at 1200 °C for 1–20 h in argon resulted in the formation of La_2_Si_2_O_7_, while the monazite was stable with SiC when the same heat treatment was done in air.

In this review, synthesis methods, crystal structures, and properties of anhydrous REPO_4_ compounds are summarized. Studies on the chemical durability and radiation stability, as well as different models for phase stability of monazite and xenotime are also summarized.

## 2 Synthesis methods

Different methods, including flux-assisted, solid state, hydrothermal, aqueous, dehydration, and gel-based methods, have been used for the synthesis of monazite and xenotime compounds. Table 1 summarizes the synthesis conditions for REPO_4_ compounds.

**Table 1:** Summary of monazite and xenotime synthesis. La → Dy* are monazite compounds, and Tb → Lu + Y, Sc are xenotime compounds. For the synthesized compound, S and P denote single crystal and polycrystalline compound. The ratio represents the mass ratio of RE oxide to flux

RE	Form	Method	Precursors	Flux	Ratio	*T* (°C)	*t*_d_ (h)	*T*_c_ (°C)	*r*_c_ (°C h^−1^)	Ref.
La	S	Flux	La_2_O_3_, PbHPO_4_	Pb_2_P_2_O_7_	1 : 24	1300	12	975	2	30
La	S	Flux	La_2_O_3_, PbHPO_4_	Pb_2_P_2_O_7_	1 : 17	1360	16	900	1	31
La	S	Hydrothermal	LaCl_3_·7H_2_O, H_3_PO_4_	—	—	140	12	—	—	32
La	S	Hydrothermal	La(NO_3_)_3_·6H_2_O, NaH_2_PO_4_	—	—	120–200	—	—	—	33
La	P	Solid state	La_2_O_3_, NH_4_H_2_PO_4_	—	—	1350	2	—	—	34
La	P	Solid state	La_2_O_3_, NH_4_H_2_PO_4_	—	—	1250	24	—	—	35
La	P	Aqueous	La_2_O_3_, H_3_PO_4_	—	—	80–200	1–48	—	—	36
La	P	Dehydration	LaPO_4_·*x*H_2_O	—	—	500	1	—	—	10
Ce	S	Flux	CeO_2_, PbHPO_4_	Pb_2_P_2_O_7_	1 : 24	1300	12	975	1	30
Ce	S	Hydrothermal	Ce(NO_3_)_3_·6H_2_O, H_3_PO_4_	—	—	140	12	—	—	32
Ce	P	Solid state	CeO_2_, NH_4_H_2_PO_4_	—	—	1350	2	—	—	34
Ce	P	Dehydration	CePO_4_·*x*H_2_O	—	—	800	1	—	—	37
Ce	P	Dehydration	CePO_4_·*x*H_2_O	—	—	600	1	—	—	10
Pr	S	Flux	Pr_6_O_11_, PbHPO_4_	Pb_2_P_2_O_7_	1 : 24	1300	12	975	2	30
Pr	P	Solid state	Pr_2_O_3_, NH_4_H_2_PO_4_	—	—	1250	24	—	—	35
Pr	P	Solid state	Pr_2_O_3_, NH_4_H_2_PO_4_	—	—	1350	2	—	—	34
Pr	P	Dehydration	PrPO_4_·*x*H_2_O	—	—	700	1	—	—	10
Nd	S	Flux	Nd_2_O_3_, KH_2_PO_4_	K_6_P_4_O_13_	1 : 4	1000	24	840	<0.2	38
Nd	S	Flux	Nd_2_O_3_, Li_2_CO_3_, MoO_3_	Li_2_Mo_2_O_7_	1 : 4	1020	720	—	<0.2	39
Nd	S	Flux	Nd_2_O_3_, PbHPO_4_	Pb_2_P_2_O_7_	1 : 24	1300	12	975	2	30
Nd	S	Flux	Nd_2_O_3_, PbHPO_4_	Pb_2_P_2_O_7_	1 : 17	1360	16	900	1	40
Nd	P	Solid state	Nd_2_O_3_, NH_4_H_2_PO_4_	—	—	1350	2	—	—	34
Nd	P	Dehydration	NdPO_4_·*x*H_2_O	—	—	700	1	—	—	10
Sm	S	Flux	Sm_2_O_3_, PbHPO_4_	Pb_2_P_2_O_7_	1 : 24	1300	12	975	<4	30
Sm	P	Solid state	Sm_2_O_3_, NH_4_H_2_PO_4_	—	—	1350	2	—	—	34
Sm	P	Dehydration	SmPO_4_·*x*H_2_O	—	—	700	1	—	—	10
Eu	S	Flux	Eu_2_O_3_, PbHPO_4_	Pb_2_P_2_O_7_	1 : 24	1300	12	975	<4	30
Eu	P	Solid state	Eu_2_O_3_, NH_4_H_2_PO_4_	—	—	1350	2	—	—	34
Gd	S	Flux	Gd_2_O_3_, PbHPO_4_	Pb_2_P_2_O_7_	1 : 24	1300	12	975	<4	30
Gd	P	Solid state	Gd_2_O_3_, NH_4_H_2_PO_4_	—	—	1350	2	—	—	34
Gd	P	Dehydration	GdPO_4_·*x*H_2_O	—	—	800	1	—	—	10
Tb*	P	Aqueous	Tb(NO_3_)_3_·*n*H_2_O, H_3_PO_4_	—	—	950	8	—	—	41
Dy*	P	Aqueous	Dy(NO_3_)_3_·*n*H_2_O, H_3_PO_4_	—	—	730	4	—	—	41
Tb	S	Flux	Gd_2_O_3_, PbHPO_4_	Pb_2_P_2_O_7_	1 : 24	1300	12	975	<4	30
Tb	P, S	Aqueous-flux	Tb(NO_3_)_3_·*x*H_2_O, NH_4_H_2_PO_4_	Na_2_CO_3_-MoO_3_	1 : 50^a^	1350	15	870	3	42
Dy	P	Dehydration	DyPO_4_·*x*H_2_O	—	—	1050	1	—	—	10
Dy	P, S	Aqueous-flux	Dy(NO_3_)_3_·*x*H_2_O, NH_4_H_2_PO_4_	NaHCO_3_-MoO_3_	1 : 50^a^	1375	144–168	870	3	43
Ho	P, S	Aqueous-flux	Ho(NO_3_)_3_·*x*H_2_O, NH_4_H_2_PO_4_	Na_2_CO_3_-MoO_3_	1 : 50^a^	1350	15	870	3	42
Er	P, S	Aqueous-flux	Er(NO_3_)_3_·*x*H_2_O, NH_4_H_2_PO_4_	NaHCO_3_-MoO_3_	1 : 50^a^	1375	144–168	870	3	43
Tm	P, S	Aqueous-flux	Tm(NO_3_)_3_·*x*H_2_O, NH_4_H_2_PO_4_	Na_2_CO_3_-MoO_3_	1 : 50^a^	1350	15	870	3	42
Yb	S	Flux	Yb_2_O_3_, PbHPO_4_	Pb_2_P_2_O_7_	1 : 24	1300	12	975	0.5	30
Lu	P, S	Aqueous-flux	Lu(NO_3_)_3_·*x*H_2_O, NH_4_H_2_PO_4_	Na_2_CO_3_-MoO_3_	1 : 50^a^	1350	15	870	3	42
Y	P, S	Aqueous-flux	Y(NO_3_)_3_·*x*H_2_O, NH_4_H_2_PO_4_	NaHCO_3_-MoO_3_	1 : 50^a^	1375	144–168	870	3	43
Y	S	Flux	Y_2_O_3_, PbHPO_4_	Pb_2_P_2_O_7_	1 : 24	1357	Several days	897	1	44
Sc	S	Flux	Sc_2_O_3_, PbHPO_4_	Pb_2_P_2_O_7_	1 : 24	1357	Several days	897	1	44

a The molar ratio of REPO_4_ to flux. The *t*_d_ is the dwell time, “*T*_c_” represents the temperature that the mixture was slowly cooled to grow single crystals, and *r*_c_ is the cooling rate.

Feigelson^30^ synthesized single crystals of LaPO_4_, CePO_4_, PrPO_4_, NdPO_4_, SmPO_4_, EuPO_4_, and GdPO_4_ monazite compounds as well as TbPO_4_ and YbPO_4_ xenotime compounds using Pb_2_P_2_O_7_ flux. The mixture of RE oxides and lead hydrogen phosphate (PbHPO_4_) with the mass ratio of 1 : 24 was placed in the Pt crucible and heated to 1300 °C at 300 °C h^−1^ and dwelled for 12 h at 1300 °C, and PbHPO_4_ was converted to lead pyrophosphate (Pb_2_P_2_O_7_) on heating. The mixture was slowly cooled to 975 °C at <4 °C h^−1^ and then naturally cooled to room temperature as the authors believed that crystallization did not proceed below 975 °C. The single crystals of monazite compounds were separated from the flux using diluted HNO_3_ solution. The monazite crystals had platelike morphologies, and the crystal sizes of LaPO_4_, PrPO_4_, and NdPO_4_ were ∼6 mm × ∼3 mm × ∼0.5 mm (the crystal sizes of other monazites were not reported). Decreasing cooling rate increased the crystal sizes of xenotime compounds. For YbPO_4_, a plate crystal with dimensions of 45 mm × 25 mm × 0.25 mm was obtained when cooled at 0.5 °C h^−1^. The single crystal or polycrystalline compound of TbPO_4_ could be prepared using TbO_2−*x*_ with PbHPO_4_ or H_3_PO_4_ respectively, and similar optical characteristics were observed from each compound. For the flux matrix, Pb_2_P_2_O_7_ was the main phase, but other phosphates including Pb_5_P_4_O_15_, Pb_4_P_2_O_9_, and/or Pb_3_P_2_O_8_ were found with loss of phosphorus after formation of REPO_4_.

Similar to Feigelson's flux method, Mullica *et al.*^31,40,45^ synthesized the single crystals of LaPO_4_, PrPO_4_, NdPO_4_, SmPO_4_, EuPO_4_, and GdPO_4_ using Pb_2_P_2_O_7_ as a flux. The mixture of RE oxides and PbHPO_4_ with the mass ratio of 1 : 17 was placed in a Pt crucible and heated to 1360 °C for 16 h. After heat treatment, the mixture was slowly cooled to 900 °C at 1 °C h^−1^ and then naturally cooled to room temperature.

Hirsch *et al.*^35^ used a solid-state method to prepare polycrystalline powders of LaPO_4_, PrPO_4_, and mixed La_1−*x*_Pr_*x*_PO_4_ monazite compounds. The appropriate amounts of RE oxides and NH_4_H_2_PO_4_ (ADP) were homogenized, pressed into pellets, placed in alumina crucibles, and heated at 1250 °C for 24 h at ambient atmosphere, and the final products were pure monazites.^35^ Perrière *et al.*^34^ used a similar method to synthesize LaPO_4_, CePO_4_, PrPO_4_, NdPO_4_, SmPO_4_, EuPO_4_, and GdPO_4_. The mixture of RE oxides and ADP was heated at 1350 °C for 2 h in air twice to make pure monazite powders.^34^

Khalili *et al.*^36^ prepared LaPO_4_ monazite, Lu_2_O_3_ xenotime, and Yb_2_O_3_ xenotime using RE_2_O_3_ and H_3_PO_4_. Powder of RE_2_O_3_ (0.002 mol) was added to round bottomed flask containing 13.7 mL of 14.6 M H_3_PO_4_ and stirred with magnetic stir for 1–24 h. The solution was diluted by adding 100 mL of water and refluxed at 130 °C for 2 h. The precipitate was filtered and washed with DIW. The sample was dried overnight and then heated to 80 °C for 1 h, and half of sample was heated at 200 °C for up to 48 h for complete dehydration. Sample heated at 80 and 200 °C were compared. The synthesized LaPO_4_ monazite was a polycrystalline powder containing some rhabdophane, and the xenotime powders were pure LuPO_4_ and YbPO_4_ compounds.

Cao *et al.*^32^ synthesized LaPO_4_ and CePO_4_ nanorods using LaNO_3_, CeNO_3_, and H_3_PO_4_ precursors. Here, 1 M La(NO_3_)_3_ or 1 M Ce(NO_3_)_3_ and 0.7 M H_3_PO_4_ were added to a solution of cetrimonium bromide in cyclohexane and *n*-pentanol. Solutions were mixed for 30 min and transferred to autoclaves. The autoclaves were heated at 140 °C for 12 h and cooled to room temperature. The crystals were washed with ethanol and DIW several times and dried in vacuum at room temperature. The obtained LaPO_4_ and CePO_4_ nanorods had about 5 μm lengths and 20–60 nm diameters.

Li and Ma^33^ synthesized LaPO_4_:Eu crystals using La(NO_3_)_3_·6H_2_O and Eu(NO_3_)_3_·5H_2_O with a molar ratio of 1 : 0.05 dissolved in DIW, and addition 5 mL of 1 M NaH_2_PO_4_ aqueous solution was done while stirring. Here, HNO_3_ or NaOH were added to adjust to a specific pH. The solution was put into an autoclave and heated at different temperature (120–180 °C). The resulting product was centrifuged and washed with DIW and dried at 60 °C for 12 h.

Wang *et al.*^39^ synthesized a single crystal of NdPO_4_ using Li_2_Mo_2_O_7_ as a flux. The Li_2_CO_3_ and MoO_3_ powders were used to prepare the Li_2_Mo_2_O_7_ flux. The mixture of Nd_2_O_3_ and Li_2_Mo_2_O_7_ with the mass ratio of 1 : 4 was placed in the Pt crucible and heated to 1020 °C for 24 h. A seed crystal was immersed in the solution and rotated at 30 rpm, and the single crystal was grown in the mixture cooling at 0.05–0.2 °C h^−1^ for ∼30 d. The resulting single crystal had the size of several millimeters.

Poitrasson *et al.*^46^ synthesized polycrystalline NdPO_4_ and GdPO_4_ compounds using a combined method of gelation and flux-assisted growth. The NdPO_4_ gel was prepared by dissolving Nd(NO_3_)_3_ and adding (NH_4_)_2_HPO_4_ solution. Precipitation of NdPO_4_ occurred while drying for several days, and the final NdPO_4_ product was obtained after briefly heating above 600 °C to remove NH_3_ and nitrates. The recovered NdPO_4_ powder was mixed with Li_2_MoO_4_ and MoO_3_ in the mass ratio of ∼2 : 1 : 1, respectively, and placed in a Pt crucible. The crucible was heated at 800 °C for 24 h, and polycrystalline NdPO_4_ powder was recovered by dissolving the flux in boiling water. A similar method was used for GdPO_4_ synthesis.

Different studies showed that monazite compounds can be easily synthesized by heat-treating rhabdophane. Jonasson and Vance^10^ showed that La-, Ce-, Pr-, Nd-, Sm-, and Gd-rhabdophane compounds converted to corresponding monazite compounds in 500–900 °C range after dehydration in 100–400 °C range. They observed that Dy rhabdophane compound converted to mixed phases of monazite and xenotime compounds at 950 °C and complete xenotime compound at 1050 °C.^10^ Adelstein *et al.*^37^ prepared CePO_4_ monazite by heating Ce rhabdophane at 800 °C for 1 h. However, a different study by Mesbah *et al.*^47^ showed that heating Nd, Eu, Gd, and Dy rhabdophane compounds at 200–500 °C converted them to NdPO_4_, EuPO_4_, GdPO_4_, and DyPO_4_ with tetragonal *P*3_1_21 space group, which is different symmetry from monazite or xenotime.

Heuser *et al.*^41^ synthesized TbPO_4_ and DyPO_4_ with monazite structures using rhabdophane of TbPO_4_·*n*H_2_O and DyPO_4_·*n*H_2_O. The rhabdophane compounds were made using a similar precipitation method by Boakye *et al.*^48^ Solutions containing RE(NO_3_)_3_·*n*H_2_O and H_3_PO_4_ were mixed while controlling the RE : P ratio and pH, and the precipitates were collected. The rhabdophane powders were heated at 200 °C for 2 h. Subsequently, Tb and Dy rhabdophane powders were heated at 950 °C for 8 h and 730 °C for 4 h, respectively, to convert to corresponding monazite compounds. The heating temperature and time were selected to avoid possible formation of xenotime structures.

## 3 Crystal structures

**Fig. 1:**
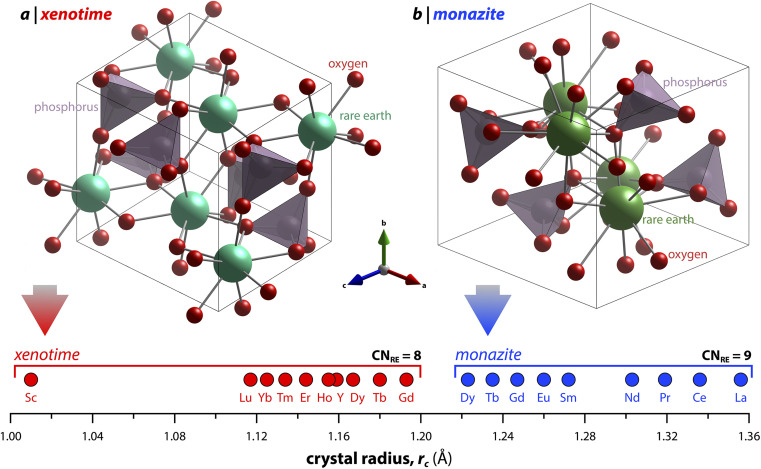
Illustration showing the relationship of (a) xenotime and (b) monazite structures of REPO_4_ to the RE crystal radii (*r*_c_) from Shannon.^49^ LuPO_4_ (ICSD 46792) and LaPO_4_ (ICSD 46788) were used to create the (a) xenotime and (b) monazite unit cell structures, respectively.

Monazite crystallizes in the monoclinic *P*2_1_/*n* space group (REs are coordinated by nine oxygens), and xenotime crystallizes in the tetragonal *I*4_1_/*amd* space group (REs are coordinated by eight oxygens) and is isostructural to zircon (ZrSiO_4_). Monazite compounds of lanthanides contain the lanthanide elements with larger ionic radii (*r*_i_) including La, Ce, Pr, Nd, Pm, Sm, Eu, Gd, Tb, and Dy whereas xenotime compounds contain the heavy lanthanide elements with smaller *r*_i_ including Gd, Tb, Dy, Ho, Er, Tm, and Lu along with Sc and Y. The structural parameters of monazite and xenotime compounds at ambient conditions (293–300 K and 1 atm) reported in the Inorganic Crystal Structure Database (ICSD) are provided in Tables 2 and 3, respectively. It should be noted that, for the REPO_4_ compounds with RE elements, the mid-range of lanthanides (*i.e.*, Gd, Tb, Dy) can crystallize in both monazite and xenotime structures. Formation of monazite or xenotime for Gd, Tb, and Dy elements can be controlled by synthesis conditions (see Section 2 for details). Fig. 1 shows the relationship of monazite and xenotime structures with respect to the crystal radii (*r*_c_) from Shannon.^49^ The unit cell parameters of the PmPO_4_ compound have been reported,^50^ but the overall structure data has not been reported in the ICSD. As for other RE elements, namely Y and Sc, YPO_4_ and ScPO_4_ (pretulite) have the xenotime structure. Both monazite and xenotime structures are commonly found in non-phosphate compounds including RE vanadates (REVO_4_) and RE arsenates (REAsO_4_).^1^ Details of the monazite and xenotime structures are discussed in the following sections.

**Table 2:** Structural parameters of REPO_4_ monazite compounds at ambient conditions (293–300 K and 1 atm) including unit cell parameters (*a*, *b*, *c*), *β* angle, cell volume (*V*), cell density (*ρ*), bond distances (*i.e.*, RE–O, P–O), and the entry number for the Inorganic Crystal Structure Database (ICSD). The space group of listed compounds is *P*2_1_/*n* (SG# 14). Volume and density are calculated values, and RE–O and P–O are the average distances

RE	*a* (Å)	*b* (Å)	*c* (Å)	*β* (°)	*V* (Å^3^)	*ρ* (g cm^−3^)	RE–O (Å)	P–O (Å)	ICSD	Ref.
La	6.7825	6.9896	6.6218	102.9602	305.92	5.08	2.5845	1.5493	46788	36
La	6.8313	7.0705	6.5034	103.27	305.73	5.08	2.5787	1.5384	79747	51
La	6.8413	7.078	6.5153	103.322	307	5.06	2.5855	1.6602	92155	52
La	6.825	7.057	6.482	103.21	303.94	5.11	2.5739	1.5350	201479	31
La	6.84133	7.07590	6.51233	103.28918	306.8	5.06	2.5729	1.5591	431743	35
Ce	6.77	7.04	6.46	104	298.74	5.23	2.6188	1.5421	22265	53
Ce	6.77	7.01	6.45	103.63	297.48	5.25	2.5467	1.5662	27860	54
Ce	6.79	7	6.46	104	297.92	5.24	2.5971	1.5154	33598	55
Ce	6.77	6.99	6.45	103.6	296.67	5.26	2.5360	1.6405	39135	56
Ce	6.77	6.99	6.45	103.63	296.63	5.26	2.5413	1.6024	43077	57
Ce	6.77	7.04	6.46	104	298.74	5.23	2.6084	1.6128	64850	53
Ce	6.7902	7.0203	6.4674	103.38	299.93	5.21	2.5586	1.5333	79746	51
Ce	6.788	7.0163	6.4650	103.43	299.49	5.21	2.5554	1.5375	79748	51
Ce	6.8072	7.00689	6.47476	103.781	299.94	5.21	2.5652	1.5318	133669	58
Ce	6.7551	6.9804	6.4687	103.707	296.33	5.27	2.5450	1.5316	133670	58
Ce	6.8004	7.0231	6.4717	103.46	300.6	5.19	2.5615	1.5397	182582	37
Ce	6.777	6.993	6.445	103.54	296.95	5.26	2.5506	1.5271	201029	59
Ce	6.78985	7.01813	6.46662	103.42415	299.72	5.21	2.5580	1.5330	243620	60
Pr	6.741	6.961	6.416	103.63	292.59	5.35	2.5323	1.5320	62161	40
Pr	6.7596	6.9812	6.4344	103.53	295.21	5.31	2.5395	1.5368	79749	51
Pr	6.77078	6.99017	6.44265	103.52914	296.46	5.28	2.5325	1.5631	431753	35
Nd	6.722	6.933	6.390	103.72	289.3	5.49	2.5192	1.5321	62162	40
Nd	6.732	6.930	6.383	103.61	289.42	5.49	2.5183	1.5369	62311	61
Nd	6.7352	6.9500	6.4049	103.68	291.31	5.45	2.5242	1.5371	79750	51
Sm	6.6818	6.8877	6.3653	103.86	284.42	5.73	2.4988	1.5370	79751	51
Sm	6.73167	6.94489	6.44964	103.899	292.7	5.57	2.5358	1.5305	133668	58
Sm	6.669	6.868	6.351	103.92	282.35	5.77	2.4932	1.5310	201839	45
Eu	6.6813	6.8618	6.3491	103.96	282.48	5.81	2.4902	1.5393	79752	51
Eu	6.639	6.823	6.318	104.00	277.69	5.91	2.4749	1.5299	201840	45
Gd	6.6435	6.8414	6.3281	103.976	279.1	6	2.4760	1.5383	79753	51
Gd	6.621	6.823	6.310	104.16	276.39	6.06	2.4693	1.5298	201841	45
Gd	6.652	6.847	6.336	103.99	280.02	5.98	2.4833	1.5373	230368	62
Gd	6.33571	6.84840	6.6516	104.023	280	5.98	2.4827	1.5325	252925	63
Tb	6.61993	6.81106	6.31653	104.1091	276.21	6.11	2.4571	1.5579	18864	41
Dy	6.59737	6.78650	6.30380	104.1887	273.63	6.25	2.4487	1.5536	18863	41

**Table 3:** Structural parameters of REPO_4_ xenotime compounds at ambient conditions (293–300 K and 1 atm) including unit cell parameters (*a*, *b*, *c*), cell volume (*V*), cell density (*ρ*), bond distances (*i.e.*, RE–O, P–O), and the entry number for the Inorganic Crystal Structure Database (ICSD). The space group of listed compounds is *I*4_1_/*amd* (SG# 141). Volume and density are calculated values, and RE–O and P–O are the average distances

RE	*a* (Å)	*b* (Å)	*c* (Å)	*V* (Å^3^)	*ρ* (g cm^−3^)	RE–O (Å)	P–O (Å)	ICSD	Ref.
Gd	6.9670	6.9670	6.1112	296.63	5.64	2.3758	1.5541	118105	64
Tb	6.9414	6.9414	6.0704	292.49	5.77	2.3726	1.5302	29316	65
Tb	6.940	6.940	6.068	292.26	5.77	2.3822	1.5228	35704	66
Tb	6.9309	6.9309	6.0606	291.14	5.79	2.365	1.5359	79755	51
Tb	6.9391	6.9391	6.0694	292.25	5.77	2.3575	1.546	168751	67
Dy	6.91	6.91	6.04	288.4	5.93	2.3469	1.548	26440	68
Dy	6.907	6.907	6.046	288.43	5.93	2.3496	1.5491	35705	66
Dy	6.9052	6.9052	6.0384	287.92	5.94	2.3513	1.5372	79756	51
Dy	6.909	6.909	6.038	288.22	5.93	2.5927	1.3327	192553	69
Ho	6.882	6.882	6.025	285.36	6.05	2.3465	1.5326	35706	66
Ho	6.8773	6.8773	6.0176	284.62	6.07	2.3428	1.5319	79757	51
Ho	6.8842	6.8842	6.0255	285.56	6.05	2.34	1.5408	246677	70
Ho	6.8919	6.8919	6.0336	286.59	6.02	2.335	1.5525	257644	71
Ho	6.886	6.886	6.027	285.78	6.04	2.3499	1.5251	257646	71
Er	6.863	6.863	6.007	282.93	6.16	2.3289	1.542	15670	72
Er	6.860	6.860	6.003	282.5	6.17	2.3361	1.5313	36052	73
Er	6.8507	6.8507	5.9968	281.44	6.19	2.3303	1.5356	79758	51
Tm	6.839	6.839	5.986	279.98	6.26	2.3252	1.5326	36053	73
Tm	6.8293	6.8293	5.9798	278.89	6.29	2.3098	1.5544	79759	51
Tm	6.8219	6.8219	5.97988	278.29	6.3	2.3238	1.5195	257645	71
Yb	6.816	6.816	5.966	277.17	6.42	2.3095	1.5439	36054	73
Yb	6.8093	6.8093	5.9639	276.53	6.44	2.313	1.5319	79760	51
Lu	6.792	6.792	5.955	274.71	6.53	2.3045	1.5327	2505	74
Lu	6.7443	6.7443	6.0105	273.39	6.56	2.2974	1.5481	46792	36
Lu	6.7827	6.7827	5.9467	273.58	6.55	2.3002	1.5337	79761	51
Lu	6.7895	6.7895	5.9560	274.56	6.53	2.3093	1.5327	162336	75
Lu	6.792	6.792	5.954	274.67	6.53	2.2998	1.5386	201133	76
Lu	6.7967	6.7967	5.9593	275.29	6.51	2.3096	1.533	246684	70
Y	6.876	6.876	6.186	292.47	4.18	2.243	1.7179	24514	77
Y	6.878	6.878	6.036	285.54	4.28	2.387	1.5365	28554	78
Y	6.9	6.9	6.026	286.9	4.26	2.2494	1.6709	56113	79
Y	6.8947	6.8947	6.0276	286.53	4.26	2.345	1.54	79754	51
Y	6.885	6.885	6.022	285.46	4.28	2.3324	1.5503	117962	80
Y	6.90706	6.90706	6.0348	287.91	4.24	2.403	1.5302	133671	58
Y	6.8817	6.8817	6.0177	284.99	4.29	2.3365	1.5435	201131	76
Sc	6.578	6.578	5.796	250.79	3.71	2.2295	1.5606	16648	81
Sc	6.5787	6.5787	5.7963	250.86	3.7	2.2116	1.5336	74483	82
Sc	6.574	6.574	5.791	250.27	3.71	2.2067	1.5341	201132	76
Sc	6.578	6.578	5.796	250.79	3.7	2.2078	1.5367	257305	83

**Fig. 2:**
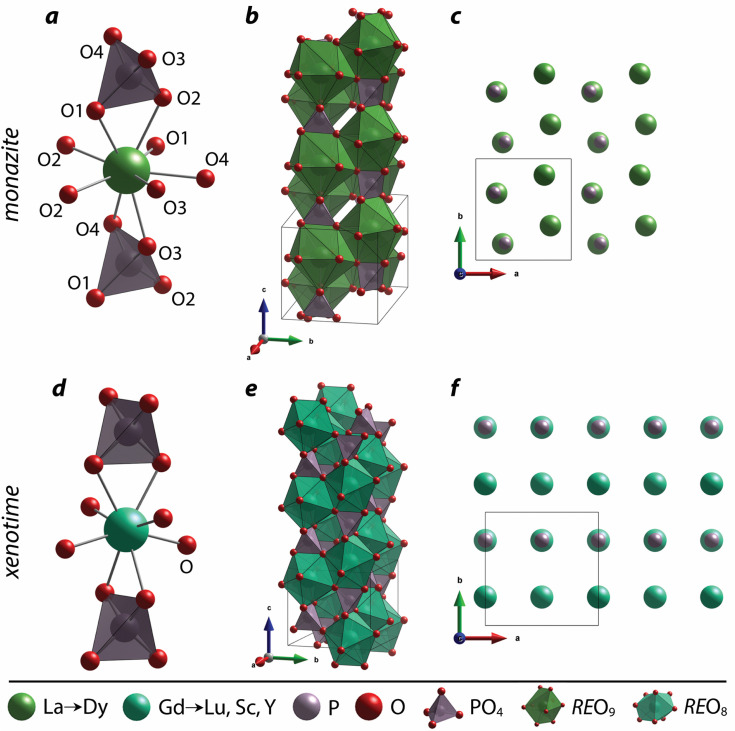
Structures of (a–c) monazite and (d–f) xenotime compounds. (a) Nine-fold coordination of the RE cation, (b) the chain of REO_9_ and PO_4_ along the *c* axis, and (c) atomic arrangements of RE and P viewed from the [001] direction in the monazite structure. (d) Eight-fold coordination of the RE cation, (e) the chain of REO_8_ and PO_4_ along the *c* axis, and (f) atomic arrangements of RE and P viewed from the [001] direction in the xenotime structure. The figure was made using CIFs of ICSD 431743 and ICSD 46792 for LaPO_4_ monazite and LuPO_4_ xenotime, respectively.

The nine oxygen atoms coordinating the REs in monazite include five oxygen atoms forming a nearly equatorial pentagon whereas the other four oxygen atoms form a tetrahedron interpenetrating the pentagon (Fig. 2), and this coordination of REO_9_ was described as a pentagonal interpenetrating tetrahedral polyhedron (PITP).^31,45^ The interpenetrating tetrahedron of REO_9_ shares the edges with two adjacent PO_4_ tetrahedra (Fig. 2a), resulting in a chain-like structure along the *c* axis (Fig. 2b). Fig. 2c shows the atomic arrangement of RE and P atoms projected down [001].

Distortion of REO_9_ polyhedra and PO_4_ tetrahedra in the monazite structure was reported in the literature.^1,59^ The monazite structure has four oxygen atom positions (*i.e.*, O1, O2, O3, and O4), and the O2 atom is shared by three RE cations and a P cation whereas O1, O3, and O4 atoms are shared by only two RE cations and a P cation. This bonding results in REO_9_ polyhedra with one longer RE–O2 distance compared to other eight RE–O distances. For example, the difference in RE–O bonding results in one longer ∼2.8 Å RE–O2 bond length compared to ∼2.5–2.6 Å of other eight RE–O bond lengths in CePO_4_.^59^ This RE-O coordination also affected the distortion of PO_4_ tetrahedra with different P–O bond lengths and O–P–O bond angles within a given PO_4_ tetrahedron.^59^ The distortion index^84^ value (*D*) can be used to show the average deviation of RE–O bond distances from their means within the REO_9_ polyhedra of monazite, and this is shown in eqn (1) where *l*_*i*_ is the distance from the central atom (*i.e.*, RE) to the *i*th coordinating atom, and *l*_av_ is the average bond length. The distortion indices of REO_9_ in monazite are in the range of 0.03–0.11 and were relatively higher than the distortion indices (0.01–0.06) of REO_8_ in the xenotime structures. The larger D value of REO_*x*_ in monazite compared to xenotime was largely due to the one long RE–O2 bond. The distorted REO_9_ polyhedron in monazite has a set of nine different RE–O bond lengths, and this coordination is correlated to its capability to incorporate various cations and polyoxoanions in the structure.^14^ Generally, this type of irregular coordination of metal ions does not induce severe symmetry, charge, or size constraints on the incorporated cation.^15,59^1
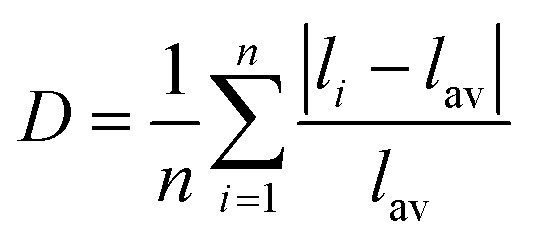


**Fig. 3:**
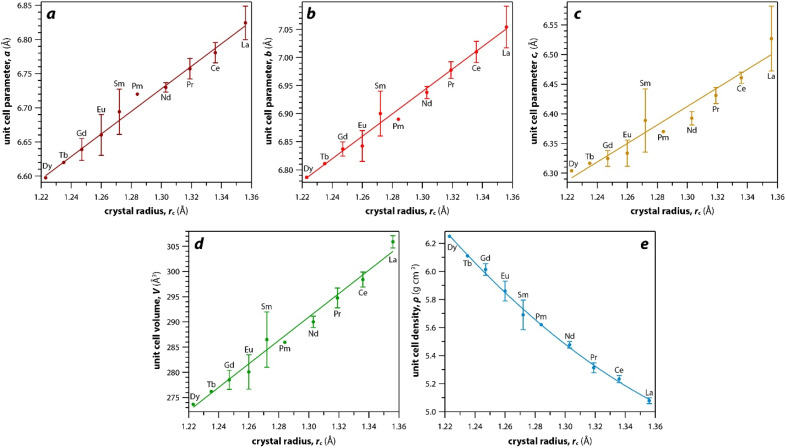
Relationships of (a–c) unit cell parameters, (d) unit cell volumes, and (e) densities with respect to RE *r*_c_ of monazite compounds. Where multiple values were given, averages and standard deviations are reported.

As mentioned earlier, the monazite structures incorporate light RE cations, including La → Dy. With larger RE cations (*i.e.*, larger *r*_c_ values)^49^ in the crystal structure, the unit cell parameters (*i.e.*, *a*, *b*, *c*) and volumes (*V*) increase linearly whereas the densities (*ρ*) decrease nonlinearly (Fig. 3). Fig. 3 was drawn using the average values of *a*, *b*, *c*, *V*, and *ρ* of monazite compounds at atmospheric conditions reported in ICSD for each given RE. The crystal structure data of PmPO_4_ was not reported in the ICSD, and the unit cell parameter values from a study by Weigel *et al.*^50^ were used to fit the trendline.

**Fig. 4:**
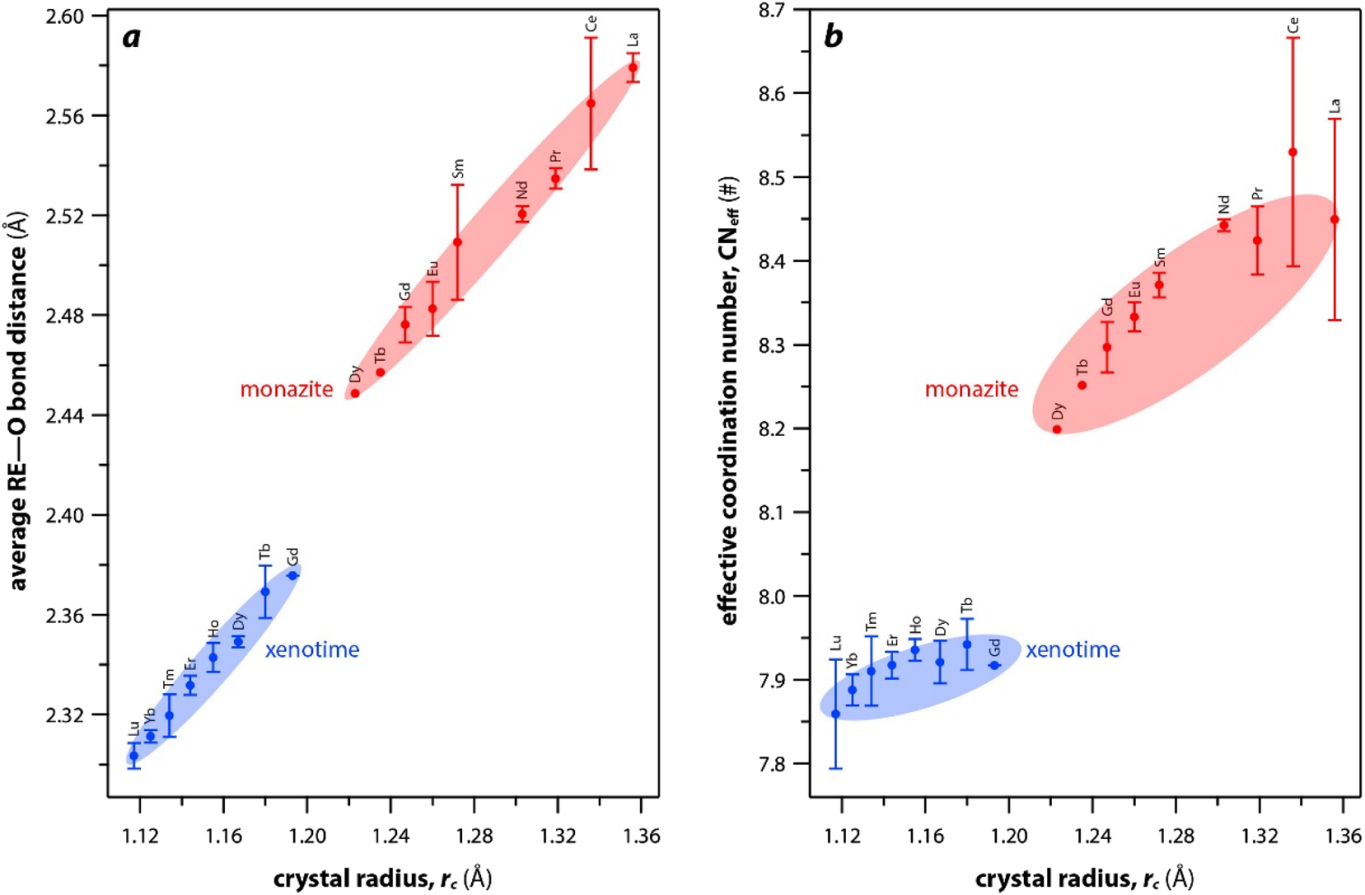
(a) Average RE–O bond distances and (b) effective coordination numbers of REO_*x*_ polyhedral with respect to RE *r*_c_ in monazite and xenotime structures.

The RE–O bond distances of monazite compounds were compared, and larger cations in REO_9_ polyhedra resulted in longer average RE–O bond distances (Fig. 4a). As discussed above, the RE cation in the monazite structure is coordinated by nine oxygen atoms, but presenting a single-digit coordination number might not be accurate in relatively distorted coordination polyhedra. The effective coordination numbers (CN_eff_)^85–87^ can be used to express more reasonable coordination numbers by accounting for all the surrounding atoms with a weighting scheme. The CN_eff_ values of REO_9_ were calculated using eqn (2) and (3) where *w*_*i*_ is the bond weight of the *i*_th_ bond, *l*_av_ is defined in eqn (4), *l*_*i*_ was defined above, and *l*_min_ is the shortest bond distance in the coordination polyhedron. These equations were formulated by combining the concepts of Pauling with effective coordination numbers and mean fictive *r*_i_ values.^87^ The average CN_eff_ value calculated for each RE element in monazites is plotted in Fig. 4b, and REO_9_ polyhedra with larger cations generally show higher CN_eff_ values.2
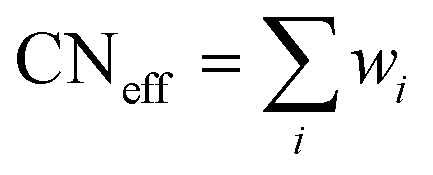
3
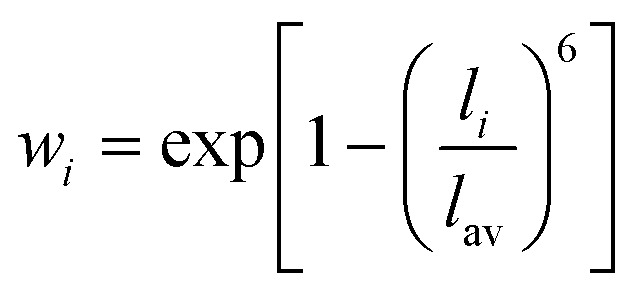
4
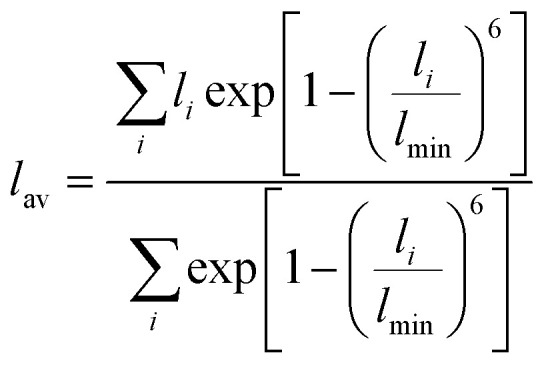


**Fig. 5:**
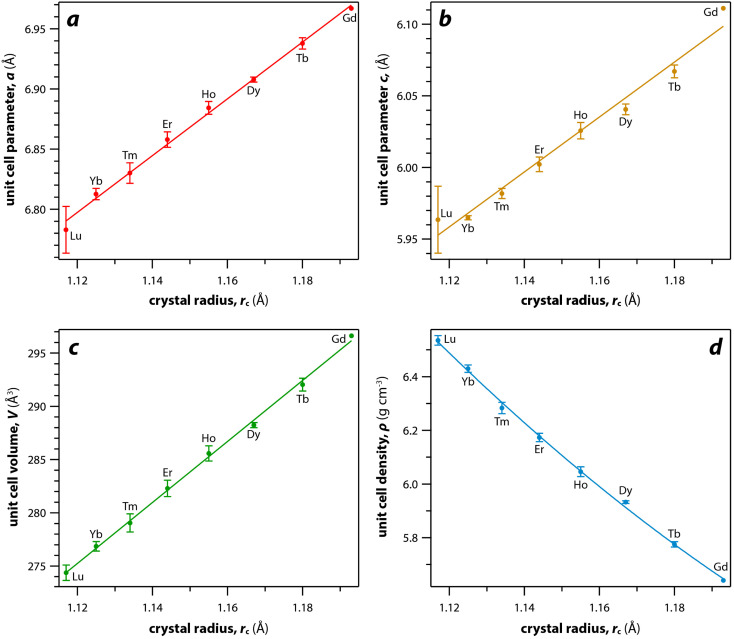
Relationships of (a and b) unit cell parameters, (c) unit cell volumes, and (d) densities with respect to RE *r*_c_ of xenotime compounds. Where multiple values were given, averages and standard deviations are reported.

For the REO_8_ coordination in xenotime, each oxygen atom is shared by two RE atoms and one P atom. Similar to monazite, the REO_8_ polyhedra share the edges with two adjacent PO_4_ tetrahedra (Fig. 2d), forming a chain-like structure along the *c* axis (Fig. 2e). Fig. 2f shows the atomic arrangement of RE and P atoms projected down [001]. With smaller RE cations in the xenotime structures compared to monazite, the D values of REO_8_ polyhedra in xenotime structures are lower than those reported for monazite structures. The xenotime compounds have shorter RE–O distances (2.2–2.4 Å) compared to monazites (2.4–2.6 Å) (Fig. 4a). The P–O bond distances of tetrahedra in xenotime compounds are generally shorter than those in monazite, but the differences are not significant. Xenotime has only one atomic position for the oxygen atom whereas monazite has four oxygen atomic positions. For both monazite and xenotime compounds, RE–P distances are dependent on the RE size, and xenotime has one RE–P distance along the chain whereas the monazite has two different RE–P distances. The RE–P distances of xenotime crystals are generally longer than those of monazite, and RE–P distances between RE^3+^ and P^5+^ cations in xenotime are generally <3 Å. The CN_eff_ values of xenotime compounds are smaller, in the range of 7.8–8.0, and smaller than monazite as expected (Fig. 4b). The unit cell parameters and volumes increase linearly with larger RE cations in the structures whereas the densities decrease nonlinearly (Fig. 5).

The RE^3+^ cations at RE sites in the monazite and xenotime structures can be substituted by mixed +3 cations, a combination of +2 and +4 cations, or a combination of +2, +3, and +4 cations. Table 4 shows the list of synthetic monazite and xenotime compounds with mixed RE and summarizes the compositional effects. For monazites with mixed RE cations, increasing the average size of mixed cations in the structures increases the unit cell parameters and volumes as expected.^88–90^ De Biasi *et al.*^88^ synthesized La_1−*x*_Ce_*x*_PO_4_ (*x* = 0–1) monazite compounds and showed that increasing La contents increased the unit cell parameters and cell volumes. Similarly, Terra *et al.*^89^ observed that increasing La contents in La_1−*x*_Gd_*x*_PO_4_ (*x* = 0–1) monazites increased the unit cell parameters and cell volumes. Thust *et al.*^91^ observed that increasing Eu content in La_1−*x*_Eu_*x*_PO_4_ (*x* = 0–1) monazite compounds increased the elastic stiffness coefficients, densities, heat capacities, and coefficients of thermal expansion (CTE). Arinicheva *et al.*^92^ observed that microhardness, fracture toughness, unit cell parameters, and cell volumes decreased linearly with increasing Eu content in La_1−*x*_Eu_*x*_PO_4_ (*x* = 0–1) monazite compounds. Van Emden *et al.*^90^ synthesized Nd_1−*x*_Y_*x*_PO_4_ (*x* = 0.05–0.3) monazite compounds using a solid state method at 1000 °C and showed that increasing Nd content increased the unit cell parameters and cell volumes. The same group also observed co-crystallization of both monazite and xenotime in Nd_1−*x*_Y_*x*_PO_4_ compounds when synthesized at 1200 °C.^90^ Hay *et al.*^93^ synthesized the Gd_1−*x*_Dy_*x*_PO_4_ (*x* = 0–1) xenotime compounds and observed that pressure and/or shear stress can cause phase transformation of xenotime to monazite. Strzelecki *et al.*^94^ investigated the thermodynamic properties of Er_1−*x*_Yb_*x*_PO_4_ (*x* = 0–1) xenotime compounds and observed that increasing Er content increased the enthalpies of formation and decreased the Gibbs free energies. The unit cell parameters and cell volumes of Er_1−*x*_Yb_*x*_PO_4_ (*x* = 0–1) increased with higher Er content as predicted by Vegard's law.^94^ Xiao *et al.*^95^ synthesized Eu^3+^-doped xenotime single crystals including TbPO_4_, HoPO_4_, ErPO_4_, YbPO_4_, LuPO_4_, and YPO_4_ with the Eu concentration of 200 ppm relative to the host RE cations. They observed that incorporation of Eu cations distorted the local structure around RE sites and affected the rotations of PO_4_ tetrahedra in the xenotime structures, and distortion levels were worse for xenotimes with large RE cations.^95^ Rafiuddin *et al.*^96^ synthesized solid solutions of La_1−*x*_Yb_*x*_PO_4_, La_1−*x*_Y_*x*_PO_4_, and Sm_1−*x*_Ho_*x*_PO_4_ (*x* = 0–1) and observed that these compounds with large differences in RE sizes resulted in the presence of both monazite and xenotime phases in the final product, and the phase fractions were dependent on the corresponding RE contents.

**Table 4:** Monazite and xenotime compounds with mixed cations and their compositional effects. M and X denote monazite and xenotime structures, respectively

Composition	Struct.	Compositional effect	Ref.
La_1−*x*_Ce_*x*_PO_4_ (*x* = 0–1)	M	Increasing La content increased the unit cell lengths and volumes	88
La_1−*x*_Gd_*x*_PO_4_ (*x* = 0–1)	M	Increasing La content increased the unit cell lengths and volumes	89
La_1−*x*_Eu_*x*_PO_4_ (*x* = 0–1)	M	Increasing Eu content increased the elastic stiffness coefficients, density, heat capacity, and CTE	91
La_1−*x*_Eu_*x*_PO_4_ (*x* = 0–1)	M	Increasing Eu content decreased the hardness, toughness, unit cell lengths, and cell volume	92
Nd_1−*x*_Y_*x*_PO_4_ (*x* = 0.05–0.3)	M	Increasing Nd content increased the unit cell parameters and cell volumes	90
Gd_1−*x*_Dy_*x*_PO_4_ (*x* = 0–1)	X	Increasing Gd content increased the maximum indentation loadings	93
Er_1−*x*_Yb_*x*_PO_4_ (*x* = 0–1)	X	Increasing Er content increased the enthalpies of formation and unit cell parameters and decreased the Gibbs free energy values	94
REPO_4_ (RE = Tb, Ho, Er, Yb, Lu, Y) doped with Eu^3+^	X	Incorporation of Eu cations distorted the local structure around RE sites and affected the rotations of PO_4_ tetrahedra, and the distortion level was worse for xenotimes with larger RE cations	95
La_1−*x*_Yb_*x*_PO_4_, La_1−*x*_Y_*x*_PO_4_, Sm_1−*x*_Ho_*x*_PO_4_ (*x* = 0–1)	M, X	Both monazite and xenotime phases were present in the final product depending on the concentration of corresponding RE elements	96

For the monazites with a mixed combination of +2 and +4 cations at RE sites, Pb, Cd, or alkaline earth metals (*e.g.*, Mg, Ca, Sr, Ba) can be incorporated with actinide cations (*e.g.*, Th, U, Np), respectively. The chemical formula is (M_*x*_^2+^)(M_(1–*x*)_^4+^)PO_4_ and many examples have been documented, including Ca_0.5_Th_0.5_PO_4_,^97^ Mg_0.5_Th_0.5_PO_4_,^98^ Sr_0.5_Th_0.5_PO_4_,^99^ Pb_0.5_Th_0.5_PO_4_,^99^ Ca_0.5_U_0.5_PO_4_,^100^ Mg_0.5_U_0.5_PO_4_,^98^ Sr_0.5_U_0.5_PO_4_,^98^ Ca_0.5_Np_0.5_PO_4_,^101^ Ca_0.5_Np_0.35_Pu_0.15_PO_4_,^102^ and Ca_0.5_Th_0.4_U_0.1_PO_4_.^103^ For the monazites with mixed combinations of +2 cations (*e.g.*, Ba, Ca, Cd, Mg, Pb, Sr), +3 cations (REs), and +4 cations (actinides) can occupy RE sites, and the site occupancies of cations with different oxidation states can vary at the RE sites (*e.g.*, Ca_1/3_Nd_1/3_U_1/3_PO_4_, La_0.808_Ba_0.096_Th_0.096_PO_4_, Ca_0.146_Nd_0.716_Th_0.151_PO_4_).^98,99,104^ The monovalent cations (*e.g.*, Li, Na, K, Rb, Cs) with +3 RE cations or Ce^4+^ can occupy the RE site of compounds that have similar structures to monazites [*e.g.*, Na_3_La(PO_4_)_2_, LiCe_2_(PO_4_)_3_, K_2_Ce(PO_4_)_2_].^105,106^

AXO_4_ monazite-type structure stabilities can be dependent on various factors such as composition, temperature, pressure, and the irradiation conditions. Clavier *et al.*^14^ reviewed crystal chemistry of the AXO_4_ monazite-type compounds in terms of field of stability *versus* composition, with all the substitution possibilities on the cationic and anionic sites leading to the monazite structure.

**Fig. 6:**
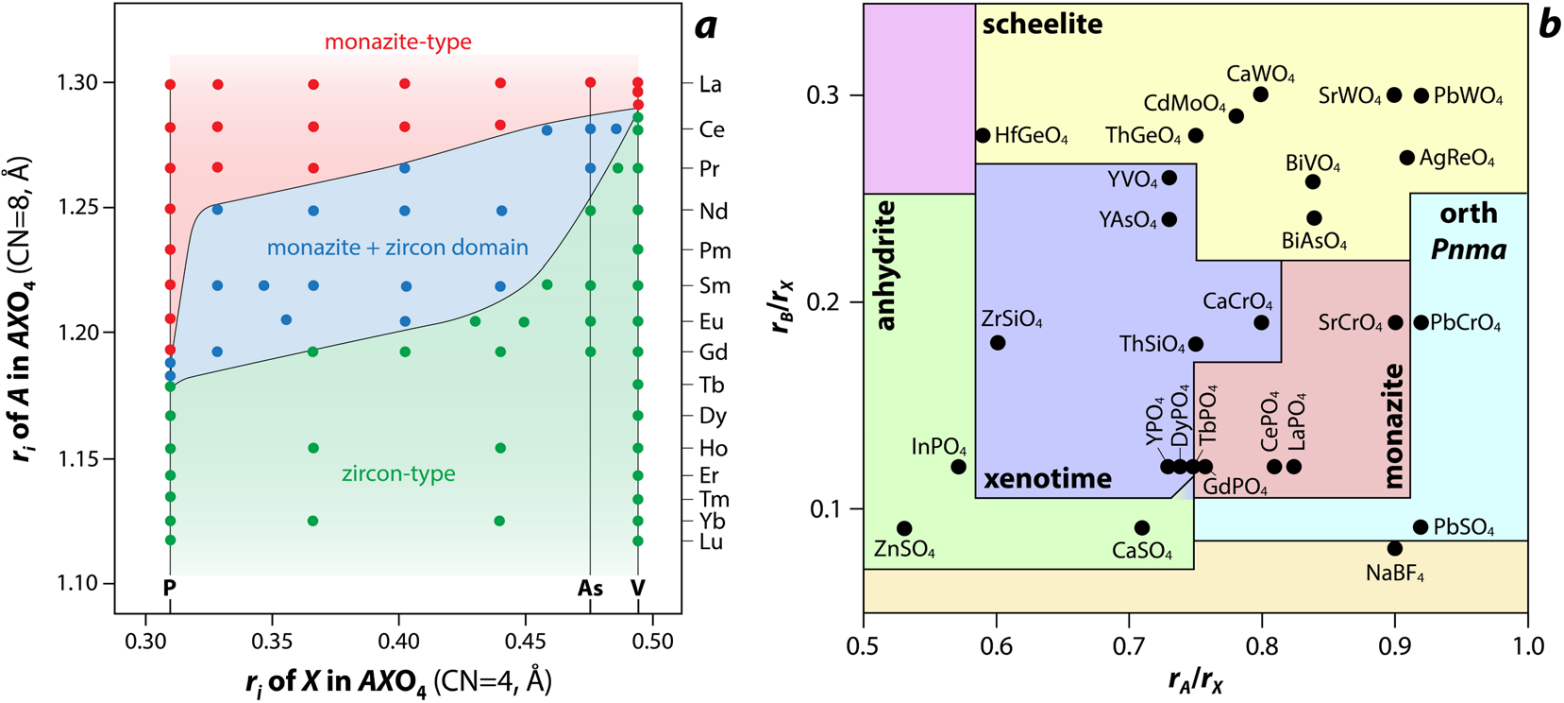
(a) Structure-field map of monazite and xenotime compounds based on the ionic radius (*r*_i_) of A (*e.g.*, RE) and X (*e.g.*, P) in AXO_4_ compounds.^107^ Reprinted with permission from Aldred.^107^ Copyright 1984 American Chemical Society. (b) Modified Bastide diagram for ABX_4_ compounds. Reprinted with permission from Hay *et al.*^115^ Copyright 2013 Elsevier.

Several models, which include structure-field maps (Fig. 6a)^107,108^ and classification diagrams (see Fig. 6b for a modified Bastide diagram),^109–112^ have been developed to correlate the stability of the monazite-type structure with geometric criteria. These representations provide opportunities to predict the structure of a compound. The challenge persists regarding the boundaries of the stability domain within this field. Numerous studies have investigated the stability domain of the monazite-type structure, aiming to develop predictive models that could anticipate whether a compound might take on the monazite structure. Carron *et al.*^113^ calculated that a value of 1.86 (ratio between the X–O bond length in the AXO_4_ compounds and the *r*_*i*_, denoted as X/*r*_*i*_) seems to specify the size limit of both the cation and the anion at the xenotime-monazite structural frontier. This ratio led to them^113^ proposing the potential for anionic isomorphous substitution among RE-phosphates, RE-silicates, RE-arsenates, and RE-vanadates. Macey^114^ further determined that monazite and zircon structures have X/*r*_*i*_ < 1/1.56 and X/*r*_*i*_ > 1/1.56, respectively.

Podor and Cuney^97^ subsequently explored the stability range of the monazite structure, primarily concentrating on (M_1–2*x*_^3+^)(M_*x*_^2+^)(M_*x*_^4+^)PO_4_ compounds, where stability depends on three parameters (*x*, average *r*_*i*_, and the ratio of M elements). Nonetheless, discrepancies with experimental observations could still occur, particularly concerning miscibility gaps. Kolitsch and Holtstam^116^ devised a visual representation outlining the stability regions of both monazite and zircon-type structures, relying on existing data for REXO_4_ (X = P, As, V) compounds. While this diagram offers a broad view of the monazite structure stability field, it is insufficient to correctly describe the partial solid solutions between two REXO_4_ compounds, especially those involving a light RE element and a heavy RE element, and the presence of a miscibility gap.^14^

## 4 Properties

### 4.1 Mechanical and thermal properties

**Fig. 7:**
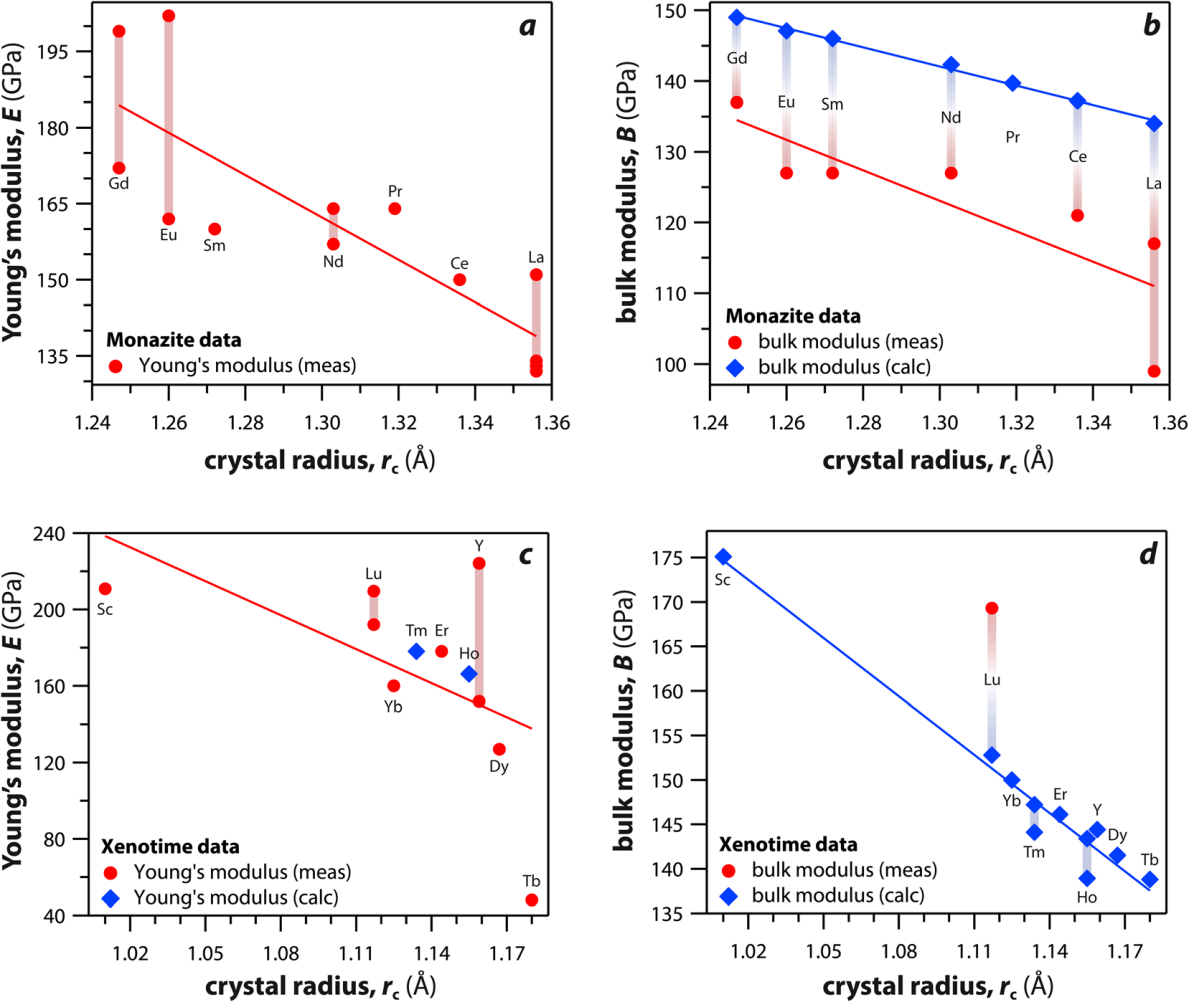
(a and c) Young's modulus and (b and d) bulk modulus values of (a and b) monazite and (c and d) xenotime compounds as functions of crystal radius of the RE cation (*r*_c_).

Monazite and xenotime compounds exhibit interesting mechanical and thermal properties across different compositions and structures. Table 5 summarizes the mechanical properties of monazite and xenotime including Young's modulus (*E*), bulk modulus (*B*), shear modulus (*G*), hardness (*H*), and flexural strength (FS). Fig. 7 shows the relationships between Young's, bulk, and shear moduli and RE *r*_c_ of monazite and xenotime. As the RE *r*_c_ values decrease, this results in increases for the Young's modulus, bulk modulus, and shear modulus, and this can be due to stronger interatomic bonding as the average RE–O distances are smaller for RE cations.^117^ Different modulus values from various studies are also affected by sample preparation and sintering conditions for the pellets.^117,118^

**Table 5:** Mechanical properties of monazite and xenotime compounds including Young's modulus (*E*), bulk modulus (*B*), shear modulus (*G*), hardness (*H*), and flexural strength (FS). M and X denote monazite and xenotime, and * denotes calculated values

RE	Struct.	*E* (GPa)	*B* (GPa)	*G* (GPa)	*H* (GPa)	FS (Mpa)
La	M	132,^118^ 133,^117^ 134,^119^ 139.1*,^120^ 144*,^119^ 151 (ref. 34)	99,^119^ 117,^117^ 107*,^119^ 109.8*,^120^ 134*^121^	51,^117^ 53,^119^ 54*,^120^ 56*,^119^ 58 (ref. 34)	4.6,^118^ 5 (ref. 122)	109,^34^ 100 (ref. 123)
Ce	M	146.5*,^120^ 150,^117^ 162*^119^	118.6*,^120^ 121,^117^ 126*,^119^ 137.2*^121^	56.6*,^120^ 58,^117^ 63*^119^	—	183 (ref. 123)
Pr	M	150.1*,^120^ 164 (ref. 34)	113.5*,^120^ 139.7*^121^	58.6*,^120^ 64 (ref. 34)	—	94 (ref. 34)
Nd	M	154.1*,^120^ 157,^117^ 164,^34^ 168*^119^	114.1*,^120^ 127,^117^ 135*,^119^ 142.3*^121^	60.4*,^120^ 61,^117^ 63,^34^ 65*^119^	—	122,^34^ 97 (ref. 123)
Pm	M	157.4*^120^	117.1*^120^	61.7*^120^	—	—
Sm	M	160,^117^ 160.3*,^120^ 172*^119^	116.2*,^120^ 127,^117^ 140*,^119^ 146*^121^	62,^117^ 63.1*,^120^ 66*^119^	—	135 (ref. 123)
Eu	M	162,^117^ 163.1*,^120^ 174*,^119^ 202 (ref. 34)	118.1*,^120^ 127,^117^ 143*,^119^ 147.1*^121^	62,^117^ 64.2*,^120^ 67*,^119^ 79 (ref. 34)	—	99 (ref. 34)
Gd	M	165.2*,^120^ 172,^117^ 180*,^119^ 199 (ref. 93)	121*,^120^ 137,^117^ 150*,^119^ 149*^121^	64.9*,^120^ 67,^117^ 69*^119^	7.8,^93^ 7.9 (ref. 124)	—
Tb	M	164.5*^120^	123.3*^120^	64.4*^120^	—	—
Dy	M	165.4*^120^	127.6*^120^	64.4*^120^	—	—
Tb	X	48 (ref. 93)	138.8*^121^	—	1.3,^93^ 5.7 (ref. 124)	—
Dy	X	127 (ref. 32)	141.5*^121^	—	4.6,^118^ 6.6 (ref. 124)	—
Ho	X	166.2*^71^	138.9*,^71^ 143.4*^121^	63.9*^71^	—	—
Er	X	178*^125^	144*,^125^ 146.1*,^121^ 168 (ref. 125)	69*^125^	—	100 (ref. 126)
Tm	X	178.1*^71^	144.1*,^71^ 147.2*^121^	68.8*^71^	—	—
Yb	X	160*^125^	129*,^125^ 150*^121^	62*^125^	—	135 (ref. 126)
Lu	X	192.1,^71^ 210*^125^	152.8*,^121^ 169.3,^71^ 170*^125^	73.3,^71^ 81*^125^	7.42 (ref. 71)	155 (ref. 126)
Y	X	145.5,^71^ 224*,^125^ 186 (ref. 127)	132.4,^71^ 144.4*,^121^ 173*^125^	55.2,^71^ 87*^125^	5.83 (ref. 71)	95 (ref. 126)
Sc	X	203,^127^ 211*^125^	175.1*,^121^ 140*^125^	84*^125^	—	—

**Fig. 8:**
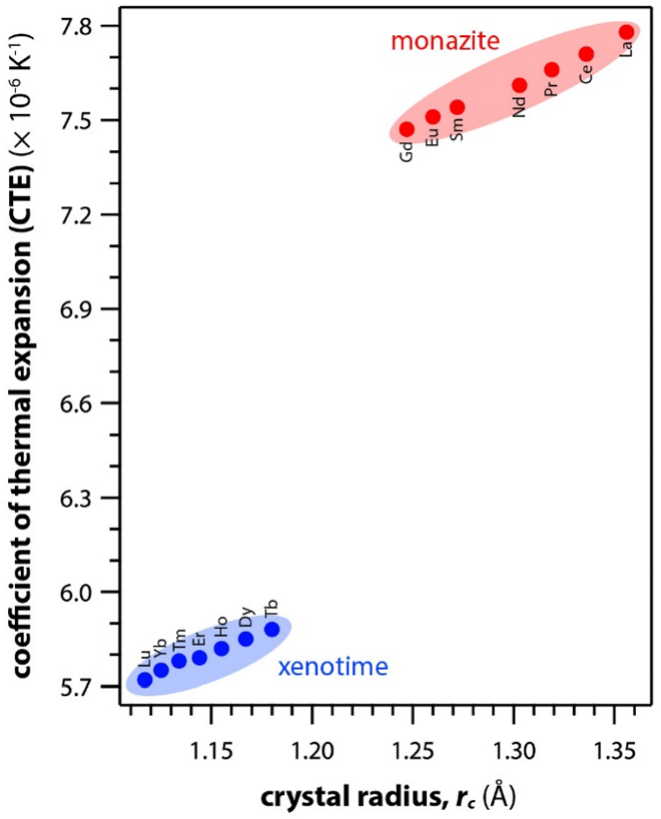
Summary of calculated CTE values for both monazite and xenotime REPO_4_ crystals from Li *et al.*^121^ as a function of RE *r*_c_.

Table 6 summarizes the thermal properties of monazite and xenotime including heat capacity (*C*_p_), the CTE, thermal conductivity (*k*), thermal diffusivity (*α*), and melting temperatures (*T*_m_). In general, the CTE values of monazite compounds seem to increase with smaller RE cations and RE–O distances in the structures. In general, the CTE values of monazite compounds seem to increase with the larger RE cations and RE–O distances in the structures. The CTE value is related to the inter-atomic potential and depends on the dissymmetry of the potential well.^34^ Examples of calculated CTE values for both monazites and xenotimes are shown in Fig. 8.^121^ The *C*_p_ values of monazite compounds are similar. The total heat capacity is the sum of the lattice component and an excess electronic term. The excess electronic heat capacity term increases for CePO_4_, PrPO_4_, NdPO_4_, SmPO_4_, and EuPO_4_ monazites but not for LaPO_4_ and GdPO_4_ due to empty and half-filled electron shell configurations, respectively, and this results in similar heat capacities among CePO_4_ → EuPO_4_, and slightly lower values for LaPO_4_ and GdPO_4_.^34,117^

**Table 6:** Thermal properties of monazite and xenotime compounds including heat capacity (*C*_p_), coefficient of thermal expansion (CTE), thermal conductivity (*k*), thermal diffusivity (*α*), and melting temperature (*T*_m_). The measured and calculated temperatures are shown in the parentheses and * denotes values that were calculated

RE	Struct.	*C*_p_ (J mol^−1^ K^−1^)	CTE (×10^−6^ K^−1^)	*k* (W m^−1^ K^−1^)	*α* (mm^2^ s^−1^)	*T*_m_ (°C)
La	M	101.28 (25 °C),^128^ 101.28* (25 °C),^128^ 106 (50 °C)^34^	7.78*,^121^ 8.2 (25–1050 °C),^118^ 8.894*,^119^ 10 (20–1000 °C),^123^ 10.3 (200–1000 °C)^34^	3.2 (25 °C),^118^ 3.61 (25 °C),^117^ 5.3 (50 °C)^34^	1.629 (25 °C)^117^	2072 (ref. 129)
Ce	M	106.4 (25 °C),^128^ 106.63* (25 °C),^128^ 110 (50 °C)^34^	7.71*,^121^ 9.029*,^119^ 9.9 (20–1000 °C)^123^	3.14 (25 °C)^117^	1.334 (25 °C)^117^	2045 (ref. 129)
Pr	M	106.04* (25 °C),^128^ 108 (50 °C)^34^	7.66*,^121^ 10.9 (200–1000 °C)^34^	3.4 (50 °C)^34^	—	1938 (ref. 129)
Nd	M	104.8 (25 °C),^128^ 104.92* (25 °C),^128^ 108 (50 °C)^34^	7.61*,^121^ 8.093*,^119^ 9.4*,^119^ 9.8 (20–1000 °C),^123^ 10.7 (200–1000 °C)^34^	3.05 (25 °C),^117^ 4.4 (50 °C)^34^	1.274 (25 °C)^117^	1975 (ref. 129)
Sm	M	105.59* (25 °C),^128^ 112 (50 °C)^34^	7.54*,^121^ 9.7 (20–1000 °C),^123^ 9.738*,^119^ 11 (200–1000 °C)^34^	2.87 (25 °C),^117^ 3.9 (50 °C)^34^	1.160 (25 °C)^117^	1916 (ref. 129)
Eu	M	110 (50 °C),^34^ 111.49 (25 °C)^128^	7.51*,^121^ 8.303*,^119^ 11.1 (200–1000 °C)^34^	2.99 (25 °C),^117^ 5.8 (50 °C)^34^	1.135 (25 °C)^117^	2200 (ref. 119)
Gd	M	102.21 (25 °C),^128^ 102.21* (25 °C),^128^ 105 (50 °C)^34^	7.47*,^121^ 8.303*,^119^ 11.4 (200–1000 °C)^34^	3.22 (25 °C),^117^ 4.8 (50 °C)^34^	1.322 (25 °C)^117^	2200 (ref. 119)
Tb	X	101.4* (25 °C)^130^	5.88*^121^	—	—	2150 (ref. 1)
Dy	X	102.5* (25 °C)^130^	5.85*^121^	—	—	2150 (ref. 1)
Ho	X	102.4* (25 °C)^130^	5.82*^121^	—	—	—
Er	X	102.3* (25 °C)^130^	5.79*,^121^ 6 (1000 °C)^126^	12.01 (20 °C)^126^	0.5 (20 °C)^126^	1896 (ref. 129)
Tm	X	102.7* (25 °C)^130^	5.78*^121^	—	—	—
Yb	X	102.8* (25 °C)^130^	5.75*,^121^ 6 (1000 °C)^126^	11.71 (20 °C)^126^	0.5 (20 °C)^126^	—
Lu	X	100.2* (25 °C)^130^	5.72*,^121^ 6.2 (1000 °C)^126^	11.97 (20 °C)^126^	0.5 (20 °C)^126^	—
Y	X	100.3* (25 °C)^130^	6.2 (1000 °C),^126^ 6.7*^121^	12.02 (20 °C)^126^	0.6 (20 °C)^126^	1995 (ref. 129)
Sc	X	—	6.95*^121^	—	—	—

Du *et al.*^117^ synthesized polycrystalline monazite compounds of LaPO_4_, CePO_4_, NdPO_4_, SmPO_4_, EuPO_4_, and GdPO_4_ and pressed into pellets using a spark plasma sintering apparatus at 1350 °C and 40 MPa for 3 min. Young's modulus increased linearly from LaPO_4_ to GdPO_4_ as the RE *r*_c_ values decreased. The specific heat values of all the monazites were similar at a given temperatures from 25 °C to 1000 °C and increased as the temperature increased except Gd monazite, which showed lower values at higher temperatures (>600 °C) compared to other monazites. The thermal conductivities of monazite compounds decreased as the temperature increased from 25 to 1000 °C, except EuPO_4_ and GdPO_4_ that showed increased thermal conductivities from 800 to 1000 °C. This is possibly due to the contribution of radiation transport effect, which becomes more important at higher temperatures. The thermal diffusivity values showed similar behavior as thermal conductivity with respect to temperature.

Perrière *et al.*^34^ investigated the structural dependence of the mechanical and thermal properties of monazite compounds including LaPO_4_, CePO_4_, PrPO_4_, NdPO_4_, SmPO_4_, EuPO_4_, and GdPO_4_. The synthesized monazite powders were pressed into pellets at 1500 °C and 65 MPa for 0.1–20 h. Both Young's modulus and shear modulus values increased with larger RE cations in the structure. The bending strengths of 94–122 MPa and fracture toughness around 1 MPa m^1/2^ showed the brittle behavior of monazite compounds, and this brittleness could cause inaccuracies when using micro-indentation analysis. The CTE values of monazites increased with smaller RE cations and RE–O distances in the structures and were in the 10–11.5 × 10^−6^ K^−1^ range. The heat capacities of all the synthesized monazites were ∼105–112 J mol^−1^ K^−1^ (with La and Gd having the lowest values). Controlling porosity during fabrication was important for achieving accurate measurements of both thermal and mechanical properties.

Li *et al.*^121^ calculated theoretical mechanical and thermal properties of monazite (La → Gd) and xenotime (Tb → Lu, Y, and Sc) compounds using the chemical bond theory of dielectric description. The CTE values increased with larger RE cations in the structures whereas the bulk moduli and lattice energies decreased. The CTE values were in the range of 7.78–7.47 × 10^−6^ K^−1^ for the La → Gd monazite compounds and 5.88–5.72 × 10^−6^ K^−1^ range for Tb → Lu xenotime compounds. The CTE and bulk modulus values were dependent on the RE–O bonds. The RE–O distances were ionically dominated and changed with different RE cations due to lanthanide contraction, and PO_4_ tetrahedra showed relatively high lattice energies and behaved nearly rigidly during deformation.

Kenges *et al.*^118^ synthesized pellet samples of LaPO_4_ monazite compounds with different sintering temperatures from 900 °C to 1500 °C and measured mechanical and thermal properties. The LaPO_4_ compounds contained a small amount of impurity phase La(PO_3_)_3_ (lanthanum metaphosophate). The LaPO_4_ monazite pellet sintered at 1100 °C showed the highest Young's modulus, toughness, and thermal conductivity. Increasing the sintering temperature increased the crystallite size and decreased the porosities of pellets. The LaPO_4_ monazite pellet sintered at 1100 °C had a Young's modulus of 132 GPa, a hardness of 4.6 GPa, a toughness of 1.6 MPa m^1/2^, a CTE of 8.2 × 10^−6^ K^−1^, and a thermal conductivity of 3.2 W m^−1^ K^−1^ at 25 °C.

Deepthi and Balamurugan^131^ compared the flexural strength and Young's modulus of LaPO_4_ and LaPO_4_ mixed with 20 mass% Y_2_O_3_. The LaPO_4_/Y_2_O_3_ pellets were sintered at 1000–1600 °C, and a pellet sintered at 1400 °C resulted in more uniform grain structure with less porosity. The LaPO_4_/Y_2_O_3_ pellet showed a reduction in flexural strength by 22% but increase of 1.05% in Young's modulus compared to the LaPO_4_ pellet.

Popa and Konings^128^ synthesized EuPO_4_ and SmPO_4_ monazite compounds and calculated their heat capacities. They used the enthalpy data of Sm and Eu monazites along with data from other La, Ce, Nd, and Gd monazites and calculated the heat capacity as a sum of lattice contributions and an excess electronic term. The calculated heat capacities of La → Gd monazites were in the range of 101.28–111.49 J mol^−1^ K^−1^ range, and these values were in good agreement with the experimental values.

Hay *et al.*^93^ investigated the phase transformations and deformation mechanisms of GdPO_4_, TbPO_4_, and DyPO_4_ compounds using scanning electron microscopy (SEM) and transmission electron microscopy (TEM) after indentation. The synthesized powders of TbPO_4_ and DyPO_4_ had xenotime structures whereas GdPO_4_ had a monazite structure. These materials were cold pressed at ∼300 MPa and sintered at 1600 °C for 20 h and 1700 °C for 1 h. The GdPO_4_, TbPO_4_, and DyPO_4_ compounds showed Young's moduli of 199, 48, and 127 GPa and hardness values of 7.8, 1.3, and 4.6 GPa, respectively. The authors observed stress-induced transformations from xenotime to monazite and suggested ferroelastic behavior under certain conditions.

Hikichi *et al.*^126^ measured the specific heats, thermal diffusivities, thermal conductivities, and bending strengths of xenotime pellets including ErPO_4_, YbPO_4_, LuPO_4_, and YPO_4_. The xenotime pellets were sintered at 700 °C to 1700 °C. The YbPO_4_ and LuPO_4_ pellets sintered above 1300 °C as well as YPO_4_ and ErPO_4_ pellets sintered above 1500 °C showed relative densities of ≥98%. The bending strengths increased with larger RE cations in the structures. The heat capacities of Er, Yb, Lu, and Y xenotime compounds were 0.40, 0.38, 0.38, and 0.48 J mol^−1^ K^−1^ at 20 °C, respectively. The CTE values were 6.0 × 10^−6^ K^−1^ for Er and Yb compounds and 6.2 × 10^−6^ K^−1^ for Lu and Y compounds.

Wilkinson *et al.*^124^ studied bulk modulus and hardness properties of EuPO_4_, GdPO_4_, TbPO_4_, and DyPO_4_ compounds using *in situ* nanoindentation for a range of loading rates and indentation depths. EuPO_4_ and GdPO_4_ formed monazite structures, and TbPO_4_ and DyPO_4_ formed xenotime structures. The bulk modulus values were decreasing in order of Gd, Eu, Dy, and Tb compounds. Both Eu and Gd compounds showed hardnesses of 8–9 GPa whereas Tb and Dy compounds showed hardnesses of 6–7 GPa. Hardnesses for all four compounds were not impacted by indentation depth or strain rate.

In addition to experimental methods, first principles calculations were also used to calculate the structural and physical properties of monazite and xenotime crystals. Kowalski and Li^120^ calculated elastic moduli of La, Ce, Pr, Nd, Pm, Sm, Eu, Gd, Tb, and Dy monazites using *ab initio* density functional theory (DFT). The Margules interaction parameters, which is related to excess enthalpy of mixing in a RE1_*x*_RE2_(1−*x*)_PO_4_ solid solution, and moduli were related to the mismatch in the endmember volumes of different RE cations within the structures. The computed Young's moduli, bulk moduli, and shear moduli increased with decreases in RE cation radii. The range of Young's moduli, bulk moduli, and shear moduli were 139–165 GPa, 110–129 GPa, and 54–65 GPa, respectively. Feng *et al.*^119^ calculated theoretical mechanical and thermal properties of monazite compounds using the results of local spin density approximation and compared to the experimental values. The Young's moduli, bulk moduli, and shear moduli of LaPO_4_, CePO_4_, NdPO_4_, SmPO_4_, EuPO_4_, and GdPO_4_ increased with smaller RE cations and shorter RE–O distances in the structures. The Young's modulus of monazite compounds showed high anisotropy. The calculated coefficients of linear thermal expansion were similar to experimental values, but the calculated thermal conductivities were higher than experimental values measured at >800 K.

Blanca-Romero *et al.*^132^ used the DFT + *U* method to calculate the structures and thermodynamic properties of monazite type crystals, with a goal to test the accuracy of the method for modeling f electron-containing systems such as RE-monazites. They found significant improvement both in terms of structures (lattice parameters, unit cell volumes, and RE–O distances for both RE oxides and phosphates) and properties (formation energies for RE phosphates and band gaps for RE oxides) of the DFT + *U* method as compared to the standard DFT in comparison to experimental values. Overall, the DFT + *U* method with the PBEsol (Perdew–Burke–Ernzerhof functional revised for solids) exchange correlation functional and Hubbard *U* parameters derived from linear response-based *ab initio* calculations was found to be a good choice for studying RE oxides and monazites.

Beridze *et al.*^133^ further investigated the DFT + *U* method for *ab initio* calculations of xenotime- and actinide-bearing complexes. The accuracy of the description of RE–O bond distances in xenotime was compared for two standard DFT xc functionals (PBEsol with f electrons in the core and f electrons in the valence shells) and the DFT + *U* (PBEsol + *U*_LR_, with the U values calculated from *ab initio* linear response). It was found that the DFT + *U* method has the best description of RE–O distances in xenotime crystals while both DFT + *U* and DFT (PBEsol with f electrons in the core) describe formation energies with good agreement with experiment, when accounting for the overestimation of P_2_O_5_ volume, similar to the case of monazites.^132^

### 4.2 Chemical durability

In general, the chemical durabilities of RE-phosphate and actinide-phosphate compounds documented in the literature are very high. Solubility product constants (*K*_sp_) for hydrated actinide-phosphate (AnPO_4_·*x*H_2_O) and RE-phosphate (REPO_4_·*x*H_2_O) compounds, which cover rhabdophane, monazite, and xenotime compounds, have been found within the range of 
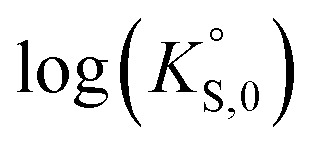
 = −27.4 to −24.5 for LaPO_4_·0.5H_2_O^134–136^ to 
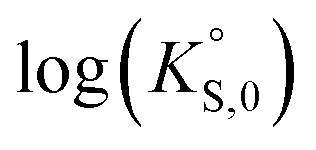
 = −66.6 for Th_2_(PO_4_)_2_(HPO_4_)·H_2_O^135,136^ at 25 °C. Values for 
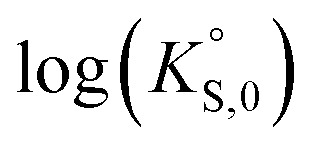
 at 100 °C were reported at −26.0 for NdPO_4_·0.5H_2_O (rhabdophane), −25.7 for PrPO_4_-0.5H_2_O (rhabdophane), and −25.5 for ErPO_4_·*n*H_2_O (xenotime).^46,134,136^ Documented apparent activation energy (*E*_a_) values fall within the 39–45 kJ mol^−1^ range.^89,137^ Oftentimes, the REPO_4_ compounds are used as surrogates for the actinide-equivalent analogs because they are easier and less costly to study than the actinide-containing phases.^138,139^ A summary of normalized release rate (NR*_i_*) data from the literature on these types of phosphate compounds is provided in Table 7.

**Table 7:** Summary of chemical durability data for REPO_4_ compounds^a^ including the composition (if given), test conditions, normalized release rate (NR_*i*_) for element “*i*” (if given), and the reference

Composition	Test conditions	NR_*i*_ (g m^−2^ d^−1^)	Ref.
Ce_0.5_Pr_0.5_PO_4_	MCC-1 (ASTM C1220); *T* = 90 °C, pH = 7, *t* = 42 days, PTFE, DIW	5.7 × 10^−6^ [Ce]	140
		5.3 × 10^−5^ [Pr]	
Natural^b^	50 °C < *T* < 229 °C; 1.62 < pH < 10.04; 50–100 μm particles	8.13 × 10^−7^ [Ce] (70 °C, pH = 6)	137
		4.13 × 10^−5^ [Ce] (70 °C, pH = 1.6)	
		9.29 × 10^−7^ [Ce] (50 °C, pH = 2)	
		2.69 × 10^−3^ [Ce] (229 °C, pH = 2)	
GdPO_4_	200 mg in 5 mL acidic solution; removed 100 μL for time resolved data points and replaced with fresh solution; 90 °C; 0.1 M HNO_3_	2.2 ± 0.7 × 10^−5^ [Gd] (RT)	89
		5.9 ± 2.1 × 10^−4^ [Gd] (90 °C)	
(La,Nd,Pr)PO_4_	Under saturation in either static tests (in HDPE with low leachate renewal) or dynamic tests (in PTFE with high leachate removal)	10^−3^ to 10^−7^	136
REPO_4_	25 °C < *T* < 90 °C	1.17 × 10^−3^ [La] (90 °C)	141
		4.3 × 10^−4^ [Ce] (90 °C)	
		1.01 × 10^−3^ [Nd] (90 °C)	
		4.02 × 10^−3^ [Gd] (90 °C)	
REPO_4_	*T* = 90 °C; 0.1 M HNO_3_	1.4 × 10^−3^ [La]	142
		1.8 × 10^−3^ [Ce]	
		1.0 × 10^−3^ [Nd]	
		1.6 × 10^−3^ [Eu]	
		1.0 × 10^−3^ [Gd]	
REPO_4_	50% H_2_O vapor balanced with O_2_ at 0.3 cm s^−1^, 1500 °C, and 1 atm for 80 h.	5.6 × 10^−2^ [Sc]	125
		6.0 × 10^−2^ [Y]	
		9.8 × 10^−2^ [Er]	
		1.0 × 10^−1^ [Yb]	
		4.5 × 10^−2^ [Lu]	

a ASTM denotes American Society for Testing and Materials International; DIW denotes deionized water; MCC denotes Materials Characterization Center; NR_*i*_ denotes the *i*-th element normalized release rate; PTFE denotes polytetrafluoroethylene; RT denotes room temperature (*i.e.*, ∼20–25 °C).

b Natural monazite from Manangotry (Madagascar) listed with composition of (Ca_0.04_La_0.21_Ce_0.43_Pr_0.05_Nd_0.15_Sm_0.02_Gd_0.01_Th_0.13_)P_0.90_Si_0.09_O_4_ with a calculated (normalized) molecular weight of 248.85 g mol^−1^.

In a study by Teng *et al.*,^140^ a mixed Ce_0.5_Pr_0.5_PO_4_ monazite sample set was produced from Pr_6_O_11_, Ce_2_(C_2_O_4_)_3_·10H_2_O, and ADP. Particles were ball milled in ethanol (4 h), dried at 60 °C (18 h), and calcined at 1000 °C (2 h). Calcined products were mixed with 5–10% polyvinyl alcohol (PVA). The calcined material was cold uniaxially pressed at 60 MPa, cold isostatically pressed at 200 MPa, and then calcined at 500 °C (6 h). Finally, samples were hot-pressed in evacuated graphite molds (<10 Pa of atmosphere, 30 MPa applied load) at temperatures of 1050–1250 °C for 0.5–4 h. A select set of samples (1150 °C for 2 h) were subjected to MCC-1 (ASTM C1220) chemical durability tests at a 1 : 10 cm^−1^ surface-area-to-volume (sample : leachant) ratio at 90 °C in PTFE containers for different testing durations ranging from 3 d to 42 d where leachates were quantitatively measured using inductively coupled plasma mass spectrometry (ICP-MS). After 42 d of leaching, the NR_*i*_ values were found to be 5.7 × 10^−6^ g m^−2^ d^−1^ for Ce and 5.3 × 10^−5^ g m^−2^ d^−1^ for Pr.

In a study by Oelkers and Poitrasson,^137^ dissolution tests were run on natural monazite from Manangotry (Madagascar) in open mixed flow reactors under different conditions of 50–229 °C (pH = 2) and 70 °C (pH = 1.6, 2.6, or 10) and closed reactors at 70 °C (pH = 2, 6, or 10). The starting material was ground to a 50–100 μm particle size fraction. The results showed a range of leach rates with the highest releases revealed for the highest test temperatures (≳200 °C).

In a study by Terra *et al.*,^89^ several monazites were made in the solid solution system of La_(1−*x*)_Gd_*x*_PO_4_ (*x* = 0, 0.2, 0.3, 0.35, 0.4, 0.5, 0.6, 0.65, 0.7, 0.8, and 1.0). Starting materials included La(NO_3_)_3_·6H_2_O, LaCl_3_·7H_2_O, Gd(NO_3_)_3_·6H_2_O, GdCl_3_·6H_2_O, and H_3_PO_4_. An excess of H_3_PO_4_ was included to prevent RE_2_O_3_ formation during synthesis. Aqueous solutions of the RE reagents (*i.e.*, 0.6–1.4 M) were prepared and these were added to 5 M H_3_PO_4_. Three different methods were used to synthesize the compounds. In the first approach, heat was applied in different steps: (1) samples were dried through direct evaporation using a sand bath, (2) dried material was ground to finer particle size and heated at 400 °C (14 h), and (3) heated at 1300 °C (10–14 h). In the second approach, the mixture was placed in a PTFE container at 150 °C in a sand bath for 1–2 weeks. The third approach utilized a hydrothermal synthesis process with additives in PTFE Parr autoclaves at 150–200 °C for 1–2 months. The GdPO_4_ compound was subjected to chemical durability testing as shown in Table 7.

In a study by Du Fou de Kerdaniel,^136^ AnPO_4_·*x*H_2_O compounds (An = Th, U) were produced using low-temperature methods based on procedures by Terra *et al.*^143^ and Clavier *et al.*^144^ or using dry chemistry routes by grinding mixtures and heating these mixtures to 1100–1400 °C where the target cations were Th, U, and REs. Leaching studies were performed under saturation in either static tests (in HDPE with low leachate renewal) or dynamic tests (in PTFE with high leachate removal) where solutions were analyzed with ICP-MS, time-resolved laser fluorescence spectroscopy, or α-scintillation counting. Dissolved solids were determined as mass loss and reported as *R*_L(*i*)_ (*i* = element of interest) where *R*_L(*i*)_ values ranged from 10^−7^ to 10^−3^ g m^−2^ d^−1^.

In a study by Hikichi *et al.*,^126^ the stabilities of RE elements of ErPO_4_, YbPO_4_, LuPO_4_, and YPO_4_ xenotime compounds in acidic or basic aqueous environments were investigated. The mass% losses of RE elements from xenotime compounds were measured using HCl, H_2_SO_4_, HNO_3_, NaOH, NH_4_OH solutions with concentrations of 6–36 N, and the test was conducted at 20 °C for 30 d. The results showed that the tested xenotime compounds were stable in these solutions, and the mass losses of RE elements were < 0.7 mass%.

In a study by Han *et al.*,^125^ xenotime powders including ErPO_4_, YbPO_4_, LuPO_4_, ScPO_4_, and YPO_4_ were cold pressed into pellets at 50 MPa and sintered at 1500 °C for 20 h in air. Chemical durability tests were performed using a vapor flow of 50% H_2_O balanced with O_2_ at 0.3 cm s^−1^ at 1500 °C and 1 atm for 80 h. The results showed dissolution rates of 5–10 × 10^−2^ g m^−2^ d^−1^ for xenotime compounds. However, the tests were performed in an alumina tube furnace, and formation of Al_5_RE_3_O_12_ compounds were observed.

Rafiuddin and Grosvenor^145^ investigated the room-temperature chemical durabilities of monazite-type, xenotime-type, and rhabdophane-type compounds on fine particles with specific surface areas ranging 1.3–15.1 m^2^ g^−1^. Two types of tests were run including dynamic tests over 84 d and static tests over 3 months. The tests revealed that the rhabdophane (GdPO_4_·H_2_O) structure released higher quantities of Gd and P ions in deionized water within the initial week of exposure. Analysis of the long-range and local structures of these materials indicates that the structures of these materials remained unchanged after seven months of leaching.

Wronkiewicz *et al.*^146^ studied the chemical durabilities of glass-crystal composites (GCCs) containing different crystalline phases, including apatite, monazite [(Ce,U)PO_4_], and spinel. The tests run included the product consistency test (PCT, *t* = 7 d, 28 d, and 91 d),^147^ the vapor hydration test (VHT),^148^ and the Toxicity Characteristic Leach Procedure (TCLP).^149^ The monazite was noted as having fewer elements (*i.e.*, Ce, U, Th) compared to the apatite sample. The leachate from the PCTs (*t* = 7–91 d) was noted as being slightly acidic (pH ≈ 5.6–6.2). The apatite and monazite samples were noted as being 100–300× more durable than a glass waste form (*i.e.*, SRL-202U) for U release.

Poitrasson *et al.*^150^ studied naturally formed magmatic monazites from European-based Paleozoic granites. Their studies showed that the hydrothermal alteration of these monazites was notably complex including a variety of mechanisms such as monoclinic → hexagonal crystal structure transitions, chemical exchanges, cation substitutions, selective Th removal, dissolution followed by precipitation, and dissolution with replacement by different minerals. They also noted that temperatures up to 300 °C likely occurred within the vicinity of the minerals.

Mikhailova *et al.*^151^ studied Pu-containing Eu-monazite and have documented Pu release being attributed to the formation of hydrated (rhabdophane) PuPO_4_ by storage in air. This calls into question the long-term disposal potential for actinides stored in REPO_4_ compounds if moisture is present in the atmosphere, which could be reduced if storage was performed in inert and dry conditions.

### 4.3 Radiation stability

The majority of naturally occurring monazites have been affected by radiation due to α decay of actinides within these minerals.^152^ This radiation has the potential to cause metamict alterations in the crystal structures of minerals, consequently elevating their solubility. In contrast to many radioactive minerals, monazite retains its crystalline structure under substantial cumulative radiation doses. Monazites are known to remain within the crystalline states by an α-healing mechanism through radiation-induced defects.^153–155^ Self-recovery of damaged structure was not observed in zircon minerals (isostructural to xenotime) with SiO_4_, but partial structural recovery was observed in xenotimes with PO_4_.^156–158^Table 8 summarizes the critical amorphization dose of monazite and xenotime compounds.

**Table 8:** Critical amorphization dose for monazite and xenotime

Materials	Critical amorphization dose	Ref.
LaPO_4_	0.15 dpa (0 K)	159
Natural monazite	7 × 10^16^ α mg^−1^	160
Natural monazite	0.13 dpa (0 K)	159
Synthetic monazite (La,Pu)PO_4_	∼(0.2–0.3) × 10^16^ α mg^−1^	161
Natural xenotime with Th and U	(1.4–14) × 10^16^ α mg^−1^	160
CePO_4_	∼0.35–0.47 dpa (∼298 K)	162
ErPO_4_ xenotime	(0.3–7.3) × 10^16^ α mg^−1^	163
Nanocrystal phosphate Rb_3_Nd(PO_4_)_2_	∼0.52 dpa	164

Meldrum *et al.*^155^ studied the effect of irradiation on monazite (LaPO_4_), xenotime (ScPO_4_), ZrSiO_4_, and ThSiO_4_ using 800 keV Kr^+^ ions. Recrystallization energies for the compounds were calculated to be 3.1–3.3 eV for the silicates and 1–1.5 eV for the phosphates. Radiation damage was monitored as a function of temperature, and above 700 °C, the amorphization of ZrSiO_4_ could not be induced as the recrystallization process was faster than damage accumulation. The critical temperature was calculated to be only 35 °C for LaPO_4_, and the monazite would not undergo phase decomposition at the tested conditions. In another study by the same group,^159^ monazite could not be amorphized when exposed to 800 keV Kr^+^ ions at temperatures surpassing 175 °C. On the contrary, zircon underwent amorphization at temperatures reaching up to 740 °C. It was found that materials with the zircon structure (*i.e.*, ZrSiO_4_ and ScPO_4_) could be amorphized at slightly elevated temperatures compared to compounds with the monazite structure under equivalent irradiation conditions.

Seydoux-Guillaume *et al.*^165^ studied the healing of radiation damage in natural monazite with annealing at 500–1200 °C. The natural monazite contained two domains with distorted lattice areas with α-dose of 2.5 × 10^16^ α mg^−1^ accumulated since 474 Ma ago. From 500–900 °C, partial healing of the lattice occurred. At 900 °C after 10 days, only one domain remained, and a well-crystallized lattice was observed. The same group performed structural analysis using X-ray diffraction (XRD), TEM, SEM, and electron probe microanalysis and showed that monazites are not metamict despite the old ages of samples ranging from 24 to 1928 Ma.^166^

Bregiroux *et al.*^167^ synthesized monazite powders containing plutonium(iii), plutonium(iv) and americium(iii). They examined the response of the monazite structure to α self-irradiation using XRD. The results revealed a total amorphization of the crystalline structure after 300 days, reaching a cumulative dose of 1.65 × 10^25^ α m^−3^.

Picot *et al.*^168^ explored the impact of Au^2+^ and He^+^ ion irradiation on monazite to simulate α-decay effects. The Au^2+^ ion-irradiation induced significant alterations in the material properties. At a damage level of 6.7 dpa, monazite displayed an approximately 8.1% increase in volume, a 59% decrease in hardness, and complete structural amorphization. Conversely, no changes in the properties of these compounds were noted following He^+^ ion implantation.

Deschanels *et al.*^169^ investigated α-induced swelling in monazite and zirconolite ceramics. It was found that the macroscopic swelling and amorphization of monazite relied on the type of irradiation. Monazite samples irradiated externally with Au became amorphous and exhibited a maximum swelling of 8%. In contrast, the swelling in samples doped with ^238^Pu was significantly smaller, at approximately 1%.

Radiuddin and Grosvenor^157^ studied the structural stabilities of La_1−*x*_Yb_*x*_PO_4_ materials implanted with Au ions. The long- and short-range order of La_1−*x*_Yb_*x*_PO_4_ (*x* = 0, 0.3, 0.7, 1.0) are influenced by ion-implantation, indicating the materials are prone to structural damage. Interestingly, in certain members of the La_1−*x*_Yb_*x*_PO_4_ series (*x* = 0.7 and 1.0), partial recovery of the structure was observed following high-dose Au ion implantation.

Sadhasivam and Rajesh^38^ studied the effect of γ-irradiation on the NdPO_4_ monazite compound using ^60^Co γ-cell source at a dose rate of 4.5 kGy h^−1^. Defect center, ionization, and charge trapping did not occur during irradiation, and no significant structural change was observed up to 150 kGy γ dose. The high level of γ dose did not affect the crystallinity and optical properties.

Rafiuddin *et al.*^163^ evaluated irradiation effects on the ErPO_4_ xenotime compound structure using high-energy dual ion-beam irradiation of 1.5 MeV Au^2+^ and 160 keV He^+^. The xenotime structure was found to undergo amorphization at a lower Au^2+^ ion-fluence than the monazite structure. Moreover, subsequential He^+^ ion-irradiation on the amorphized ErPO_4_ samples did not lead to the structural restoration of xenotime. Simultaneous ion-irradiation of Au^2+^ and He^+^ prevented the amorphization of ErPO_4_ as higher amounts of electronic energy was applied, similar to the α-healing mechanism in the monazite structures where high energies cause recrystallization faster than damage accumulation. However, the α-healing mechanism for xenotime required ∼4 times more energy compared to monazite.

Overstreet *et al.*^170^ investigated the structural stability of SmPO_4_ and TbPO_4_ under swift heavy ion irradiation using 1.1 GeV ^197^Au ions. Both SmPO_4_ monazite and TbPO_4_ xenotime structures experienced amorphization at comparable rates with increasing fluence, and complete amorphization occurred ∼5 × 10^12^ ions cm^−2^ for both compounds. No irradiation-induced recrystallization was observed at higher fluences for both compounds. Findings from this study differed from other radiation damage using relatively low-energy ions, where the monazite compounds were more resistant to amorphization compared to xenotime compounds. The results from this study suggested that the crystal chemistries and structures of monazite and xenotime will not greatly affect the radiation tolerance to highly energetic ions.

Tisdale *et al.*^164^ synthesized single crystals of Rb_3_RE(PO_4_)_2_ (RE = Y, La, Pr, Nd, and Sm→Lu) by high-temperature flux growth methods. The 1.2 MeV Xe^3+^ ions were used on the Rb_3_Nd(PO_4_)_2_ sample to investigate the radiation effect, and complete amorphization was observed by 0.22 dpa for a single crystal sample and ∼0.52 dpa for a polycrystalline sample. DFT calculations were performed for trivalent actinide analogs of Rb_3_M(PO_4_)_2_ (M = Am, Cm), and the results indicated high tolerance to radiation damage.

Burakov *et al.*^161^ studied radiation resistance effects, including amorphization, of different crystalline host phases on Pu storage to simulate the effects of long-term disposal. The study included Pu-doped cubic zirconia (Zr_0.79_Gd_0.14_Pu_0.07_O_1.99_), monazites [(La,Pu)PO_4_, PuPO_4_, and (Eu,Pu)PO_4_], zircon [(Zr,Pu)SiO_4_], and pyrochlore [(Ca,Gd,Hf,Pu,U)_2_Ti_2_O_7_]. The (La,Pu)PO_4_ and PuPO_4_ monazites remained crystalline until receiving cumulative doses of 1.19 × 10^25^ α m^−3^ and 4.2 × 10^24^ α m^−3^.

### 4.4 Optical properties and applications

Several studies have documented the study of optical properties of REPO_4_ compounds, including optical spectroscopy^171,172^ and Raman spectroscopy.^173–175^ Hernández and Martín^172^ studied the ultraviolet-visible-near-infrared (UV-Vis-NIR, *i.e.*, 200–3000 nm) absorption spectra of EuPO_4_. Absorption spectra show low to medium absorption within the *λ* = 400–1700 nm region of the spectrum (Vis-NIR) but high absorptions in the UV (*λ* < 400 nm) and NIR (*λ* > 1700 nm). They noted that the absorption spectra remained unchanged, even after 18 kGy irradiation with ^60^Co. Studies have shown the utility of doped REPO_4_ compounds to function as scintillators when exposed to X-rays or γ-rays.^1^ Examples of these types of materials include LuPO_4_:Ce,^176^ LuPO_4_:Nd,^177^ YPO_4_:Nd,^177^ as well as Sm-doped or Eu-doped YPO_4_, ScPO_4_, and LuPO_4_.^178^ REPO_4_ compounds can also be used as thermophosphors to provide a remote-sensing probe for temperature determination, which was demonstrated using LuPO_4_: (Dy,Eu).^24,25^ An example of where this would have utility is where it is not practical to use metal thermocouples, *e.g.*, remote sensors within a microwave environment.

## 5 Future work and perspectives

After considering the wealth of data collected on the wide range of anhydrous REPO_4_ compounds discussed within this paper, more work is needed to fill in research gaps summarized within this section. Additional thoughts and perspectives are provided throughout where future work could be done.

Regarding mechanical properties, extensive datasets are available for *E*, *B*, and *G* while several experimental gaps exist in datasets for *H* and FS (see Table 5). Many of the datasets from single studies do not correlate well with data from other studies or measured data fall far from calculated data (see Fig. 7), leading to difficulty in making accurate predictions in some cases. Gaps exist in the thermal properties for the different series (Table 6) and the data spread across literature values for single properties of a given REPO_4_ compound tends to be rather high in some cases. For one dataset of calculated CTE values, the spread was very low (see Fig. 8), but that was anomalous against the entire set of summarized data. Two of the most populated datasets include *C*_p_ and CTE. More work needs to be done to better elucidate the variabilities in these properties across the full RE dataset.

Since APO_4_ compounds are promising as stable options for long-term disposal of radionuclides, including rare earths and actinides, more detailed studies on their chemical durabilities are needed. The normalized release rates (NR_*i*_) for tested REPO_4_ compounds show very low values (Table 7) as compared to other nuclear waste forms, such as borosilicate glass. Understanding potential incongruent elemental release from these compounds is important, including comparisons between NR_P_ and NR_RE_ over long time scales under dilute conditions.

While some radiation stability tests have been documented in the literature for REPO_4_ compounds (see Table 8 for examples), additional studies are needed. This includes wider compositional ranges as well as more high-energy exposure studies (*e.g.*, γ-rays). Several studies have demonstrated amorphization doses for a variety of REPO_4_ compounds and types of irradiations. A study by Nasdala *et al.*^179^ provided evidence that α-assisted annealing can prevent irradiation-induced amorphization in CePO_4_ monazite, but only above a specific damage level. Understanding how REPO_4_ compounds behave in potential geological repository environments and the transition of REPO_4_ compounds to the hydrated (*i.e.*, rhabdophane or REPO_4_·*x*H_2_O) forms is very important from a waste form perspective for long-term disposal.

An overview of DFT-based first principles calculations on mechanical, structural, and thermodynamic properties was briefly covered due to the effectiveness of the method to predict the structures and properties of this class of materials. It was found that the usage of the DFT + *U* method^180,181^ to treat f electrons in RE elements was essential to improve the description of both structures and properties. More work can be done to study defect formation energies and mixing of RE elements in REPO_4_ compounds using first principles methods. First principles calculations can also be used to study high-entropy monazite-based or xenotime-based ceramics formation and properties through mixing of various RE elements. In addition, classical molecular dynamics (MD) simulations can be used to study radiation effects and thermomechanical behaviors.^182^ Recent advances using machine learning potentials^183^ based on first principles calculations can help to alleviate the bottleneck of interatomic potential availability. However, extensive testing and validation of these new potentials will be needed. Combining these techniques, one can expect computational methods to provide valuable information regarding the distribution of RE elements within the crystal lattice, the role of dopants in controlling properties, radiation induced structural change and amorphization, and the mechanisms governing phase transformations under extreme conditions.

With the rapid development of applying machine learning techniques in material science,^184^ the integration of predictive models and machine learning holds transformative potential across various applications of REPO_4_ compounds.^185–187^ These models can be utilized to predict properties such as phase stability, mechanical behavior, and chemical reactivity under various/extreme conditions. Future efforts, including constructing a more consistent dataset including trace elements, developing high-throughput parallel simulation routines, and standardizing characterization methods, would greatly benefit the implementation of machine learning in studying monazite and xenotime composition–structure–property relationships. By training models on experimental and/or simulation data, researchers can extrapolate insights beyond the limits of traditional analysis, guiding experimental design and hypothesis generation.

## 6 Summary and conclusions

The synthesis methods, crystal structures, and properties of anhydrous monazite and xenotime crystalline materials are summarized within this review. Monazite and xenotime compounds can be synthesized with a variety of different methods including flux-assisted, solid state, hydrothermal, aqueous, dehydration, and gel-based methods. For both monazite and xenotime structures, with larger and lighter RE cations in the crystal structure, the unit cell parameters (*i.e.*, *a*, *b*, *c*) and volumes (*V*) increase linearly whereas the densities (*ρ*) decrease nonlinearly. Similar trends were observed for solid solution compounds containing mixed RE cations. Some solid solutions containing RE cations with large difference in sizes (*e.g.*, La and Yb) showed the presence of both monazite and xenotime phases. For RE cations, the distortions of REO_*x*_ polyhedra were greater in monazites when compared to xenotimes. Decreasing RE radii increased the Young's modulus, bulk modulus, and shear modulus, and this can be due to stronger interatomic bonding as the average RE–O distances decreased with smaller RE cations. Different modulus values from different studies are also affected by different sample preparation and sintering conditions for the pellets. In general, the CTE values of monazite compounds seem to increase with the larger RE cations and RE–O distances in the structures. The calculated CTE values usually followed the trends, but the experimental CTE values from different studies often varied greatly, and it was difficult to create a general trendline. First-principles DFT calculations have been shown to be a reliable predictive method for both the structures and properties of monazite and xenotime crystals, although care was needed in the description of the f-electrons and the DFT + *U* method was found to be reliable and computationally feasible approach for this purpose. The chemical and radiation resistance of monazite and xenotime are similar to that of zircon, and thus the natural minerals are often used in geochronology. Monazite compounds are generally more resistant to irradiation damage compared to the xenotime compounds.

## Conflicts of interest

There are no conflicts to declare.

## Supplementary Material
